# Genome-wide CRISPR screen identifies synthetic lethality between DOCK1 inhibition and metformin in liver cancer

**DOI:** 10.1007/s13238-022-00906-6

**Published:** 2022-02-26

**Authors:** Junru Feng, Hui Lu, Wenhao Ma, Wenjing Tian, Zhuan Lu, Hongying Yang, Yongping Cai, Pengfei Cai, Yuchen Sun, Zilong Zhou, Jiaqian Feng, Jiazhong Deng, Ying Shu, Kun Qu, Weidong Jia, Ping Gao, Huafeng Zhang

**Affiliations:** 1 Anhui Key Laboratory of Hepatopancreatobiliary Surgery, Department of General Surgery, Anhui Provincial Hospital, The First Affiliated Hospital of USTC, Division of Life Science and Medicine, University of Science and Technology of China, Hefei, 230027 China; 2 Hefei National Laboratory for Physical Sciences at Microscale, The Chinese Academy of Sciences Key Laboratory of Innate Immunity and Chronic Disease, School of Basic Medical Sciences, Division of Life Science and Medicine, University of Science and Technology of China, Hefei, 230027 China; 3 Shenzhen People’s Hospital, Second Clinical Medical College of Jinan University, First Affiliated Hospital of Southern University of Science and Technology, Shenzhen, 518000 China; 4 Department of Pathology, School of Medicine, Anhui Medical University, Hefei, 230032 China; 5 School of Medicine and Institutes for Life Sciences, South China University of Technology, Guangzhou, 510006 China

**Keywords:** CRISPR screen, DOCK1, hepatocellular carcinoma, metformin, small GTPase

## Abstract

Metformin is currently a strong candidate anti-tumor agent in multiple cancers. However, its anti-tumor effectiveness varies among different cancers or subpopulations, potentially due to tumor heterogeneity. It thus remains unclear which hepatocellular carcinoma (HCC) patient subpopulation(s) can benefit from metformin treatment. Here, through a genome-wide CRISPR-Cas9-based knockout screen, we find that DOCK1 levels determine the anti-tumor effects of metformin and that DOCK1 is a synthetic lethal target of metformin in HCC. Mechanistically, metformin promotes DOCK1 phosphorylation, which activates RAC1 to facilitate cell survival, leading to metformin resistance. The DOCK1-selective inhibitor, TBOPP, potentiates anti-tumor activity by metformin* in vitro* in liver cancer cell lines and patient-derived HCC organoids, and* in vivo* in xenografted liver cancer cells and immunocompetent mouse liver cancer models. Notably, metformin improves overall survival of HCC patients with low DOCK1 levels but not among patients with high DOCK1 expression. This study shows that metformin effectiveness depends on DOCK1 levels and that combining metformin with DOCK1 inhibition may provide a promising personalized therapeutic strategy for metformin-resistant HCC patients.

**Supplementary Information:**

The online version contains supplementary material available at 10.1007/s13238-022-00906-6.

## Introduction

Liver cancer represents a fourth common cause of cancer-related deaths worldwide (Bray et al. 2018). Sorafenib and lenvatinib are the first-line drugs in targeted therapies for advanced unresectable HCC (Llovet et al. 2008; Kudo et al. 2018). However, high toxicity and the development of drug resistance limits the respective clinical benefits of these targeted therapies. Several recent studies have explored the mechanisms of drug resistance in order to identify new therapeutic strategies for HCC (Sun et al. 2016; Qiu, Li et al. 2019; Wei et al. 2019; Jin et al. 2021). For example, treatment with the combination of lenvatinib plus gefitinib resulted in meaningful clinical response in HCC patients with high EGFR levels (Jin et al. 2021). Large-scale genome sequencing and multi-omics analyses revealed that liver cancer is highly heterogeneous (Ally 2017; Jiang et al. 2019), suggesting that therapeutic strategies may be limited to subpopulations of patients. Therefore, more effective drugs or personalized therapeutic strategies for advanced HCC are urgently needed.

Metformin is currently the most widely used anti-diabetic drug, and has a favorable safety profile at relatively low cost (Nathan et al. 2009). Several recent studies have indicated that metformin potentially reduces cancer incidence as well as cancer-related mortality rates associated with various human malignancies including breast, liver and pancreatic cancers (Chen et al. 2013; Pernicova and Korbonits 2014). These studies have subsequently led to increased interest in the mechanisms by which metformin acts as an anti-tumor agent, and other studies have shown that metformin exhibits anti-tumor effects through multiple, complex and inter-related metabolic and signaling pathways. For example, metformin was shown to directly inhibit the mitochondrial electron transport chain, thereby decreasing ATP levels and activating AMPK (Quinn et al. 2013; Vancura et al. 2018). Activation of AMPK subsequently inhibits fatty acid synthesis and gluconeogenesis (Zhou et al. 2001; Li et al. 2011), ultimately inhibiting tumorigenesis. In addition to metabolic regulation, metformin-activated AMPK also inhibits tumor progression through epigenetic remodeling (Galdieri et al. 2016). AMPK can also directly phosphorylate tuberous sclerosis complex protein 2, leading to accumulation of RHEB-GDP and inhibition of mTORC1 activation (Inoki et al. 2003; Gwinn et al. 2008), resulting in decreased protein synthesis and cellular growth. Moreover, metformin treatment also decreases reactive oxygen species levels and inhibits the cell cycle in an AMPK-independent manner (Bonnefont-Rousselot et al. 2003; Ben Sahra et al. 2011).

However, with an increasing body of evidence showing the activity of metformin in a range of cellular processes, inconsistencies in the anti-tumor effects of metformin have also arisen, suggesting that these effects may be limited to certain subpopulations. For example, a clinical metabolomics study revealed that metformin contributes to diverse metabolic responses in primary breast cancer patients, leading to distinct anti-tumor effects (Lord et al. 2018). In addition, Lee et al. showed that BATCH1 deficiency resulted in enhanced anti-tumor activity by metformin in triple-negative breast cancer, while targeting BATCH1 with hemin led to metformin sensitivity in these cells (Lee et al. 2019). Another recent study also revealed that metformin selectively inhibits metastatic colorectal cancer carrying the KRAS mutation but not the wild type (Xie et al. 2020), which further suggested that the anti-tumor effects of metformin vary in different cancer subpopulations. However, the specific subtypes of liver cancer that are sensitive to metformin treatment remain unknown, thus limiting the potential for application of metformin in liver cancer patients as an anti-tumor agent. The inherent heterogeneity of liver cancer thus presents an urgent need for a systematic screen to identify biomarkers that can predict the therapeutic response to metformin and help determine the subpopulations of liver cancer patients that could benefit from metformin treatment.

Here, we used a CRISPR-Cas9-based negative selection strategy followed by correlation analysis between drug response and gene expression levels to systematically identify genes whose loss resulted in enhanced anti-tumor effects under metformin treatment in liver cancer. Interestingly, we identified a previously undescribed role for DOCK1 in determining the therapeutic effectiveness of metformin against HCC. Specifically, we revealed that DOCK1 inhibition by shRNAs or TBOPP, a DOCK1 selective inhibitor, suppressed metformin-induced RAC1 activation, thus enhancing the anti-tumor effects of metformin in both patient-derived organoids and mouse models of liver cancer. Collectively, our findings demonstrate that DOCK1 levels largely determine whether metformin will be an effective therapy for individual HCC patients, and that DOCK1 targeting is a promising strategy for sensitizing liver cancer cells to metformin.

## Results

### CRISPR-Cas9 library screening identifies DOCK1 as a determinant for metformin sensitivity

**Figure 1:**
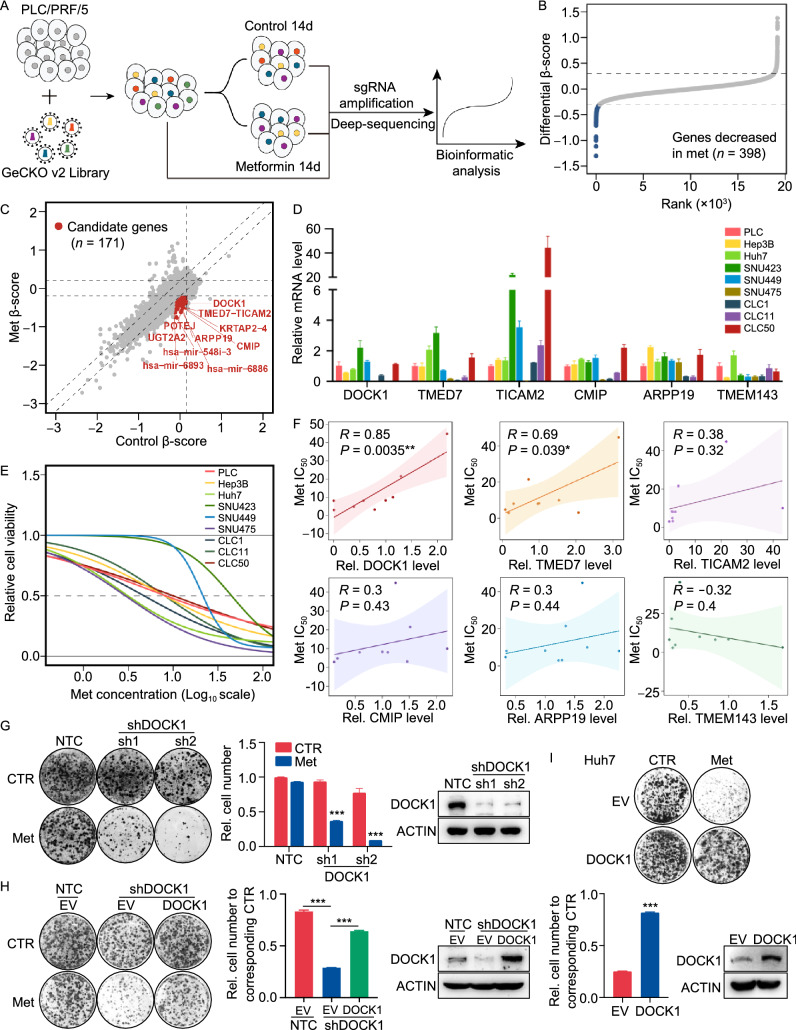
CRISPR-Cas9 library screening identifies DOCK1 as a determinant for metformin sensitivity. (A) Schematic of the synthetic lethal screen. (B) Distribution of differential beta-score (metformin treatment versus control) for genes. Blue dots indicate genes decreased in metformin-treated group (*n* = 398). (C) Scatter plot of beta-score in control and metformin-treated group. Red dots represent genes decreased only in metformin-treated group (*n* = 171). Top 10 genes are selected based on the differential beta-score and labelled. (D) qPCR analysis of the mRNA expression of six candidate genes in nine liver cancer cell lines. (E) IC_50_ curves of metformin in nine liver cancer cell lines. (F) Correlation between gene expression and metformin IC_50_. Each data point represents a liver cancer cell line. The solid line was fit from linear regression. The shading represents the confidence interval. (G) and (H) Colony formation assay were performed in the indicated PLC cell lines with or without 1 mmol/L metformin treatment (Left). Cell number were quantified (Middle). DOCK1 levels were determined by Western blot (Right). (I) Colony formation assay in the indicated Huh7 cell lines with or without 2 mmol/L metformin treatment (Top). Cell number were quantified and DOCK1 levels were determined by Western blot (Bottom). For (D), (G), (H), (I), data are presented as mean ± SD

To systematically identify subtypes of liver cancer that are sensitive to metformin treatment, we employed a CRISPR-Cas9-based negative selection approach to screen for genes whose loss potentiates the anti-tumor effects of metformin. PLC/PRF/5 cells (PLC) transfected with lentivirus containing a pooled genome-scale CRISPR knockout library (GeCKO v2) were cultured in the presence or absence of metformin. Following a two-week incubation, genomic DNA was isolated and high throughput sequencing was used to determine the abundance of guide RNA prior to further analysis by MAGeCKFlute (Fig. 1A). The results indicated that most genes, as well as non-targeting control sgRNAs, showed similar scores between the control and metformin treatment groups (Fig. S1A). However, 398 genes were significantly decreased in the metformin-treated group (differential beta-score < −0.3) (Fig. 1B). To identify which of these genes potentially sensitized the PLC cells to metformin but exhibited no obvious growth impairment in the untreated cells, we narrowed the candidates using the additional criteria of a beta-score variation in the control group of no more than 0.15 (−0.15 < Control beta-score < 0.15). Following this criterion, 171 of the 398 genes were identified as candidates (Fig. 1C), and we subsequently focused on the top 15 most depleted genes (Fig. S1B), ultimately selecting six of these for further analysis.

Next, to verify the screening results, we quantified the mRNA expression of these six candidates, and also determined the half-maximal inhibitory concentrations (IC_50_) values of metformin in nine kinds of liver cancer cells (Fig. 1D and 1E). Correlation analysis revealed that DOCK1 expression had the highest Pearson correlation coefficient with IC_50_ values and AUC scores of metformin (Figs. 1F and S1C), which suggested that DOCK1 levels may contribute to determining the sensitivity of liver cancer cells to metformin. Colony formation experiments confirmed the differences in response to metformin by the nine kinds of liver cancer cells (Fig. S1D). Given the high differential beta-score and its strong correlation with metformin response, we focused on DOCK1 for further investigation.

Analysis of the guide RNA revealed that all of six sgRNAs targeting DOCK1 had lower abundance in metformin-treated cells (Fig. S1E). Consistent with these results, DOCK1 knockdown resulted in substantial sensitization of PLC cells in both long-term colony formation assays and in IC_50_ detection to analyze short-term cell viability (Figs. 1G and S1F). Similar results were observed in SNU449 cells (Fig. S1G). Notably, ectopically expressed DOCK1 attenuated shDOCK1-induced metformin sensitivity in PLC cells (Fig. 1H). Further, overexpression of DOCK1 eliminated the anti-tumor effects of metformin in Huh7 cells (Fig. 1I). Taken together, these results indicated that DOCK1 expression levels determine the sensitivity of liver cancer cells to metformin.

### Inhibition of DOCK1 sensitizes liver cancer cells to metformin *in vivo* and *in vitro*

**Figure 2:**
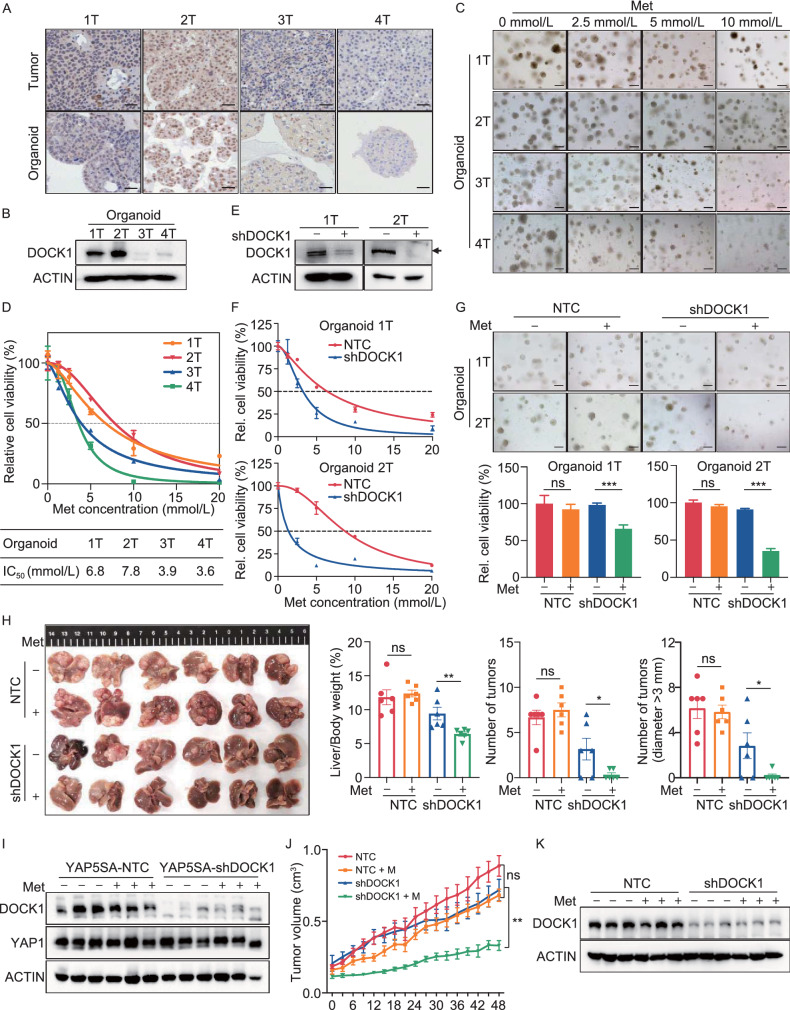
Inhibition of DOCK1 sensitizes liver cancer cells to metformin *in vivo* and *in vitro*. (A) Representative images of DOCK1 in HCC tissues and organoids by immunohistochemical staining. Scale bar, 50 µm. (B) Western blot analysis of DOCK1 expression in four HCC organoids. (C) Representative micrographs of organoids treated with indicated dose of metformin for 6 days. Scale bar, 100 µm. (D) IC_50_ curves of metformin in four HCC organoids. (E) Western blot analysis of DOCK1 expression in Organoid 1T and 2T stably expressing NTC or shDOCK1. (F) IC_50_ curves of metformin in Organoid 1T and 2T stably expressing NTC or shDOCK1. (G) Organoid 1T and 2T stably expressing NTC or shDOCK1 were treated with or without metformin (2 mmol/L for Organoid 1T and 2.5 mmol/L for Organoid 2T) for 6 days. Representative micrographs (Top) and relative cell viability (Bottom) were analyzed. Scale bar, 100 µm. (H) Plasmids expressing YAP5SA-NTC or YAP5SA-shDOCK1 together with plasmids expressing PB transposase were delivered into mice by hydrodynamic injection following with or without 100 mg/kg metformin treatment (*n* = 6 in each group). Tumor images, Liver-to-body ratio, number of tumors, and number of tumors (diameter > 3 mm) were measured at the end of the experiment. (I) Western blot analysis of DOCK1 and YAP1 expression in tumor tissues of each group described in Fig. 2H. (J and K) PLC xenografts were treated with or without 100 mg/kg metformin (*n* = 5 for each group). Tumor sizes (J) and DOCK1 expression (K) were measured. For (D), (F), and (G), data are presented as mean ± SD. For (H) and (J), data are presented as mean ± SEM

To further characterize the role of DOCK1 in determining metformin sensitivity in pre-clinical models, we established four patient-derived HCC organoids (i.e., 1T, 2T, 3T, and 4T) for further *in vitro* analyses. Histological analysis confirmed that these HCC organoids retained the histological features of the original tumors (Fig. S2A). Further immunohistochemical staining revealed consistent expression of DOCK1 in all four organoids and their corresponding tumor tissues (Fig. 2A). Both immunohistochemical staining and Western blot showed that organoids 1T and 2T exhibited significantly higher DOCK1 expression than that of 3T and 4T (Fig. 2A and 2B).

To investigate the response to metformin by HCC organoids, we treated the four organoids with increasing doses of metformin. Interestingly, organoids 3T and 4T showed a greater decrease in organoid number and size under metformin treatment than 1T and 2T (Fig. 2C), indicating higher sensitivity, which was confirmed by lower IC_50_ values for 3T and 4T (Fig. 2D). To study whether DOCK1 expression indeed determines metformin sensitivity in patient-derived organoids, we knocked down DOCK1 by shRNAs in organoids 1T and 2T. Consistent with our observation in PLC cells, DOCK1 knockdown induced metformin sensitivity in these patient-derived HCC organoids (Fig. 2E–G). Moreover, immunofluorescence staining of Ki67 revealed that metformin strongly inhibited the proliferation of HCC organoid 2T under DOCK1 knockdown (Fig. S2B). Taken together, these results documented that the DOCK1 expression levels contribute to determining the metformin sensitivity of patient-derived HCC organoids.

To investigate whether these* in vitro* findings could be recapitulated* in vivo*, we employed a YAP5SA-induced HCC model (Guo et al. 2017). To establish this model, plasmids expressing YAP5SA and shDOCK1 (or non-targeting control, NTC) in a single transposon were injected into mice by hydrodynamic injection. Following one month of growth, a low dose of metformin (100 mg/kg) was orally administered to these mice daily for three months. In the NTC group, no effects on tumor growth were observed following the low dose of metformin, whereas the incidence of liver cancer significantly decreased and tumor growth was suppressed by this metformin dose in the shDOCK1 group (Fig. 2H). Consistent with these results, Ki67 immunohistochemical staining revealed that metformin treatment markedly inhibited the proliferation of HCC tumors expressing shDOCK1 (Fig. S2C). Western blot analysis confirmed the overexpression of YAP1 and knockdown of DOCK1 in tumor tissues (Fig. 2I). In addition, mouse xenograft experiments using PLC cells with DOCK1 knockdown showed that suppression of DOCK1 resulted in potentiation of the anti-tumor effects of metformin (Figs. 2J, 2K, and S2D). Collectively, these results indicated that DOCK1 levels regulate the strength of metformin’s anti-tumor effects on liver cancer, while suppression of DOCK1 sensitizes liver cancer to metformin in both patient-derived HCC organoids *in vitro* and mouse models *in vivo*.

### RAC1 activation contributes to DOCK1-mediated cancer cell insensitivity to metformin

**Figure 3:**
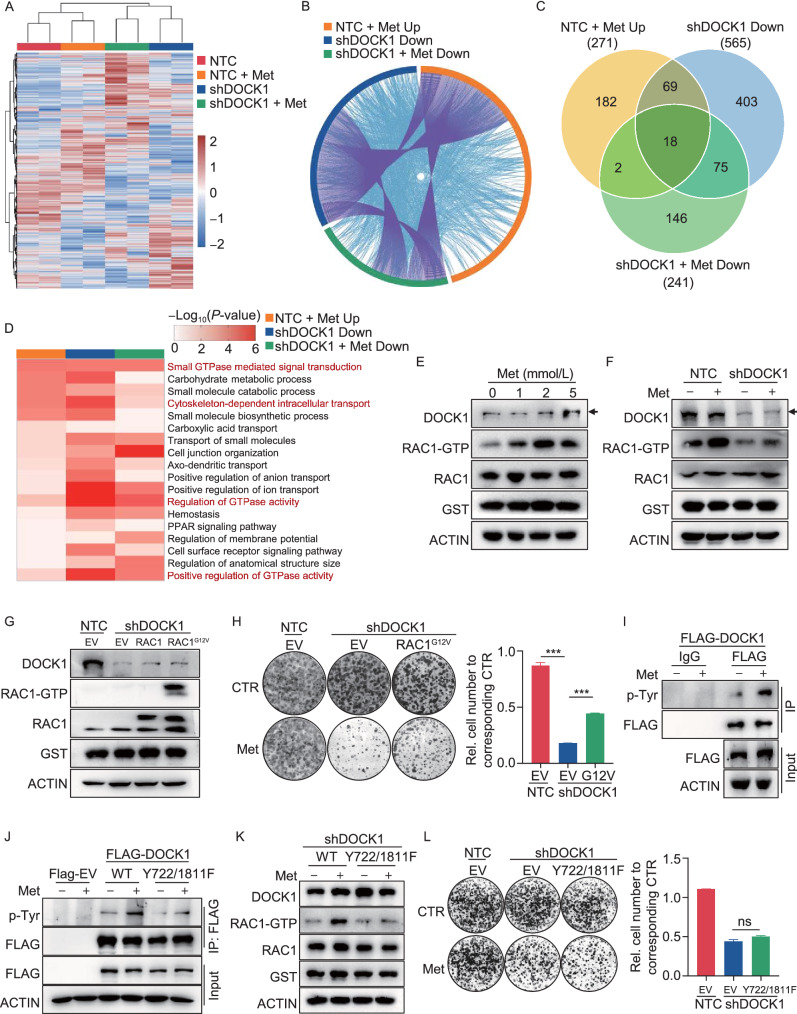
RAC1 activation via DOCK1 phosphorylation contributes to metformin insensitivity in cancer cells. (A) Heatmap of RNA-seq analysis, colors indicate the z-scored normalized counts. (B) The Circos plot shows the overlap of genes in three groups. Purple lines link the same gene that are shared by multiple groups, blue lines link the different genes where they fall into the same ontology term. NTC + Met Up: NTC + Met/NTC > 1.5; shDOCK1 Down: shDOCK1/NTC + Met < 0.5; shDOCK1 + Met Down: shDOCK1 + Met/NTC + Met < 0.5. (C) Venn diagram shows the overlap of GO terms enriched in each group. (D) Heatmap of 18 GO terms overlapped in Fig. 3C. (E) Western blot analysis of DOCK1, RAC1 expression and RAC1-GTP level in PLC cells treated with 0 mmol/L, 1 mmol/L, 2 mmol/L, and 5 mmol/L metformin for 48 h. (F) PLC-NTC and PLC-shDOCK1 cells were further cultured with or without 2 mmol/L metformin for 48 h, followed by Western blot analysis of DOCK1, RAC1 expression and RAC1-GTP level. (G) Western blot analysis of DOCK1, RAC1 expression and RAC1-GTP level in the indicated PLC cell lines. (H) Colony formation assay in the indicated PLC cell lines with or without 1 mmol/L metformin treatment (Left). Cell number were quantified (Right). (I) PLC cells stably expressing Flag-DOCK1 were treated with or without 2 mmol/L metformin for 48 h, followed by immunoprecipitation using anti-Flag or IgG antibody. Pan-phospho-tyrosine were analyzed by Western blot. (J) PLC cells stably expressing Flag-EV, Flag-DOCK1, Flag-DOCK1^Y722/1811F^ were treated with or without 2 mmol/L metformin for 48 h, followed by immunoprecipitation using anti-Flag antibody. Pan-phospho-tyrosine were analyzed by Western blot. (K) PLC cells stably expressing shRNA targeting the 3′UTR of DOCK1 were further infected with viruses expressing Flag-DOCK1 or Flag-DOCK1^Y722/1811F^ . Cells were treated with or without 2 mmol/L metformin for 48 h. DOCK1, RAC1 expression and RAC1-GTP level were analyzed by Western blot. (L) Colony formation assay in the indicated PLC cell lines with or without 1 mmol/L metformin treatment (Left). Cell number were quantified (Right). Data are presented as mean ± SD

To explore how DOCK1 deficiency enhances the anti-tumor effects of metformin, we performed RNA-seq analysis in PLC cells expressing NTC or shDOCK1 (PLC-NTC, PLC-shDOCK1 cells) in the presence or absence of metformin. We found that the expression of 935 genes was altered by metformin in NTC cells, some of which were further affected by DOCK1 suppression (Fig. 3A). To comprehensively interpret the role of DOCK1 in metformin-mediated cancer inhibition, we analyzed three sets of genes, including the metformin-upregulated genes in NTC cells, and genes that were down-regulated by shDOCK1 in the presence or absence of metformin relative to metformin-treated NTC cells. Interestingly, we observed substantial overlap in function among these three sets of genes (Fig. 3B). Further Gene Ontology (GO) and pathway enrichment analysis revealed 18 GO terms or pathways that were shared in common by all three groups (Fig. 3C). Notably, among the 18 GO terms, four terms were related to small GTPase activity (Fig. 3D), which suggested that the pathway for small GTPase activity may be involved in DOCK1 suppression induced sensitization of cancer cells to metformin.

Considering that DOCK1 is a canonical guanine nucleotide exchange factor (GEF) for RAC family small GTPases which can promote the activation of RAC (Kiyokawa et al. 1998; Brugnera et al. 2002), we focused on the RAC family small GTPase signal transduction pathway. Similar to other GTPases, RAC is active when bound to GTP and inactive when bound to GDP. Indeed, our data showed that knockdown of DOCK1 markedly reduced the level of RAC1-GTP, i.e., the active form of RAC1 (Fig. S3A). Since RAC1 has been shown to play a major role in cytoskeleton assembly, tumorigenesis and tumor proliferation, we thus hypothesized that DOCK1 deficiency sensitizes cancer cells to metformin via inhibition of RAC1 activation.

Western blots suggested that metformin treatment led to the activation of RAC1 in PLC cells, indicated by increased levels of RAC1-GTP in the presence of metformin (Fig. 3E). Moreover, metformin-mediated RAC1 activation was eliminated in DOCK1 knockdown cells, which suggested that DOCK1 was necessary for RAC1 activation by metformin (Fig. 3F). Previous studies have demonstrated that the DOCK homology region-2 (DHR2) domain of DOCK1 directly interacts with nucleotide-free RAC to induce GTP loading of RAC, thereby promoting its activation. Thus, deletion of the DHR2 domain induces loss of function in DOCK1 (Côté and Vuori 2002). To further clarify whether shDOCK1 sensitized cancer cells to metformin via its canonical GEF function, we ectopically expressed DOCK1 carrying a DHR2 domain deletion (DOCK1^△DHR2^) in PLC cells with endogenous DOCK1 knockdown. Our data showed that DOCK1^△DHR2^ had no effect on metformin sensitivity by cancer cells (Fig. S3B), which demonstrated that the GEF function is required by DOCK1 to mediate metformin sensitivity.

To further test whether RAC1 activation was essential for shDOCK1-mediated metformin sensitization, we overexpressed either wild-type RAC1 or a RAC1^G12V^ mutant, a constitutively active form of RAC1 (Xu et al. 1994), in PLC-shDOCK1 cells (Fig. 3G). Colony formation assays revealed that RAC1^G12V^ expression partially attenuated the enhanced metformin sensitivity in PLC-shDOCK1 cells, whereas wild-type RAC1 showed only negligible effects in PLC-shDOCK1 cells (Figs. 3H, and S3C), which indicated that RAC1 activation contributes to shDOCK1-mediated metformin sensitization for cancer cells.

Subsequent quantitative real-time PCR (qPCR) and Western blot analyses showed that metformin exhibited no effects on either the RNA or protein expression of DOCK1 (Figs. 3E and S3D). However, our results showed that metformin treatment resulted in enhanced phosphorylation of DOCK1 at tyrosine residues (Fig. 3I), which has been reported to increase its GEF activity (Feng et al. 2011, 2012). In order to identify which specific DOCK1 tyrosine residue(s) were phosphorylated during exposure to metformin, we constructed a DOCK1^Y722F/Y1811F^ plasmid harboring Y722F and Y1811F double mutants. Western blot showed that metformin-induced phosphorylation of tyrosine was markedly reduced in the DOCK1^Y722F/Y1811F^ variant compared to that in wild-type DOCK1 (Fig. 3J), which suggested that DOCK1 Y722 and Y1811 residues are indeed the phosphorylation sites regulated by metformin. Similar results were obtained using these constructs in 293T cells (Fig. S3E).

Moreover, we ectopically expressed wild-type DOCK1 or DOCK1^Y722F/Y1811F^ in PLC-shDOCK1 cells. Western blot showed that metformin treatment promoted the activation of RAC1 in cells expressing wild-type DOCK1, but not in cells expressing the DOCK1^Y722F/Y1811F^ mutant, thus demonstrating that RAC1 activation by metformin requires phosphorylation of DOCK1 at the Y722 and Y1811 residues (Fig. 3K). It is noteworthy that co-expression of the DOCK1^Y722F/Y1811F^ mutant also failed to attenuate shDOCK1-induced sensitization of cancer cells to metformin treatment (Figs. 3L and S3F). Taken together, these results demonstrated that metformin functions in the phosphorylation of DOCK1, resulting in the activation of RAC1, and consequently, deficiency of DOCK1 sensitizes cancer cells to metformin.

### Synergistic effects of TBOPP and metformin both *in vivo* and *in vitro*

**Figure 4:**
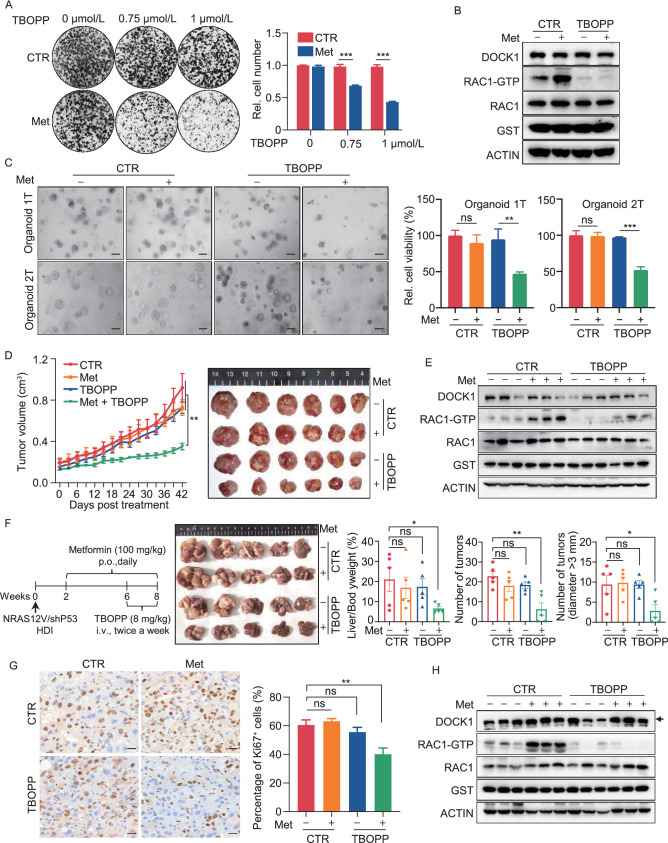
Synergistic effects of TBOPP and metformin both *in vivo* and *in vitro*. (A) Synergistic response to the combination of TBOPP and metformin in PLC cells (Left). Cell number were quantified (Right). (B) Western blot analysis of DOCK1, RAC1 expression and RAC1-GTP level in PLC cells with indicated treatment for 48 h. (C) Organoid 1T and 2T were treated with DMSO, metformin (2 mmol/L for Organoid 1T and 2.5 mmol/L for Organoid 2T), TBOPP (1.5 µmol/L for Organoid 1T and 7.5 µmol/L for Organoid 2T) or combined therapy for 6 days. Representative micrographs (Left), and relative cell viability (Right) were analyzed. Scale bar, 100 µm. (D) and (E) PLC xenografts were treated with 100 mg/kg metformin (p.o., daily), 8 mg/kg TBOPP (i.v., twice a week) or a combined therapy (*n* = 6 for each group). Tumor sizes and images were measured (D). The expression of DOCK1, RAC1 and the level of RAC1-GTP of each group were analyzed by Western blot (E). (F) Plasmids expressing NRAS^G12V^, shP53 together with plasmids expressing SB transposase were delivered into mice by hydrodynamic injection (*n* = 5 in each group). Metformin and TBOPP was administered to mice as indicated (Left). Tumor images, liver-to-body ratio, number of tumors, and number of tumors (diameter > 3 mm) were measured at the end of the experiment. (G) Representative Ki67 staining (Left) and quantified data (Right) in tumor tissues described in Fig. 4F. Scale bar, 50 µm. (H) Western blot analysis of DOCK1, RAC1 expression and RAC1-GTP level in tumor tissues described in Fig. 4F. For (A) and (C), data are presented as mean ± SD. For (D), (F) and (G), data are presented as mean ± SEM

1-(2-(30-(trifluoromethyl)-[1,10-biphenyl]-4-yl)-2-oxoethyl)-5-pyrrolidinylsulfonyl-2(1H)-pyridone (TBOPP) has recently been identified as a selective inhibitor of DOCK1 (Tajiri et al. 2017). To explore the therapeutic potential of targeting the DOCK1-RAC1 axis, we tested the effects of TBOPP on metformin toxicity in cancer cells. Our results indicated that TBOPP significantly inhibited RAC1 activation at doses of 0.75 µmol/L or 1 µmol/L, but showed no effect on PLC cell viability (Fig. S4A and S4B). However, when PLC cells were given a combined treatment of 1 mmol/L metformin with 0.75 µmol/L or 1 µmol/L TBOPP, we observed a strong synergistic effect in reducing cell viability (Fig. 4A). Similar results were observed in SNU449 and Hep3B cells (Fig. S4C). Western blot detection of RAC1-GTP revealed that metformin-induced RAC1 activation was attenuated by TBOPP in PLC cells (Fig. 4B). Subsequently, we detected IC_50_ values of 8.95 µmol/L and 31.08 µmol/L in response to TBOPP treatment in organoids 1T and 2T, respectively (Fig. S4D). We therefore used 1.5 µmol/L and 7.5 µmol/L TBOPP in combination with metformin for treatment of organoids 1T and 2T, respectively. The results showed that metformin or TBOPP alone only slightly inhibited growth and proliferation of patient-derived HCC organoids, while their combined treatment significantly decreased cell viability in both HCC organoids (Fig. 4C), suggesting that a potent, synergistic lethality accompanied the combination of metformin and TBOPP.

We next performed mouse xenograft experiments using PLC cells. Xenograft-bearing mice were treated with 8 mg/kg or 16 mg/kg TBOPP by intravenous injection twice a week, and the results showed that 8 mg/kg of TBOPP inhibited the activation of RAC1 but exhibited only modest suppression of tumor growth and mouse body weight (Fig. S4E–H). Thus, we selected an 8 mg/kg TBOPP dose in subsequent experiments to explore its combined effects with metformin. Notably, 8 mg/kg TBOPP with 100 mg/kg metformin significantly suppressed PLC xenograft tumor growth without affecting mouse body weight, and further confirming the anti-tumor synergism between TBOPP and metformin (Figs. 4D and S4I). Western blot analysis of tumor tissue lysates revealed that TBOPP treatment obviously suppressed the metformin-induced RAC1 activation (Fig. 4E), which confirmed that RAC1 activation contributes to the DOCK1 suppression associated sensitization of cancer cells to metformin* in vivo*.

To further corroborate the synergistic effect between TBOPP and metformin, we employed an NRAS^G12V^/shP53-induced orthotopic HCC model (Carlson et al. 2005; Wiesner et al. 2009). Similar to our observations in the PLC xenograft models, metformin or TBOPP monotherapy provided only modest tumor inhibition, while the combination treatment resulted in strong tumor inhibition in the NRAS^G12V^/shP53-induced mouse HCC model (Fig. 4F). We used Ki67 staining to further confirm the differences between treatments in tumor proliferation (Fig. 4G), and Western blot showed that TBOPP abolished metformin-mediated RAC1 activation in the NRAS^G12V^/shP53-induced orthotopic HCC model (Fig. 4H). Collectively, our data demonstrated that treatment with TBOPP, a DOCK1 inhibitor, results in a strong synergistic effect with metformin in suppressing liver cancer in several models both *in vitro* and *in vivo*.

### DOCK1 levels determine the anti-tumor activity of metformin in liver cancer patients

**Figure 5:**
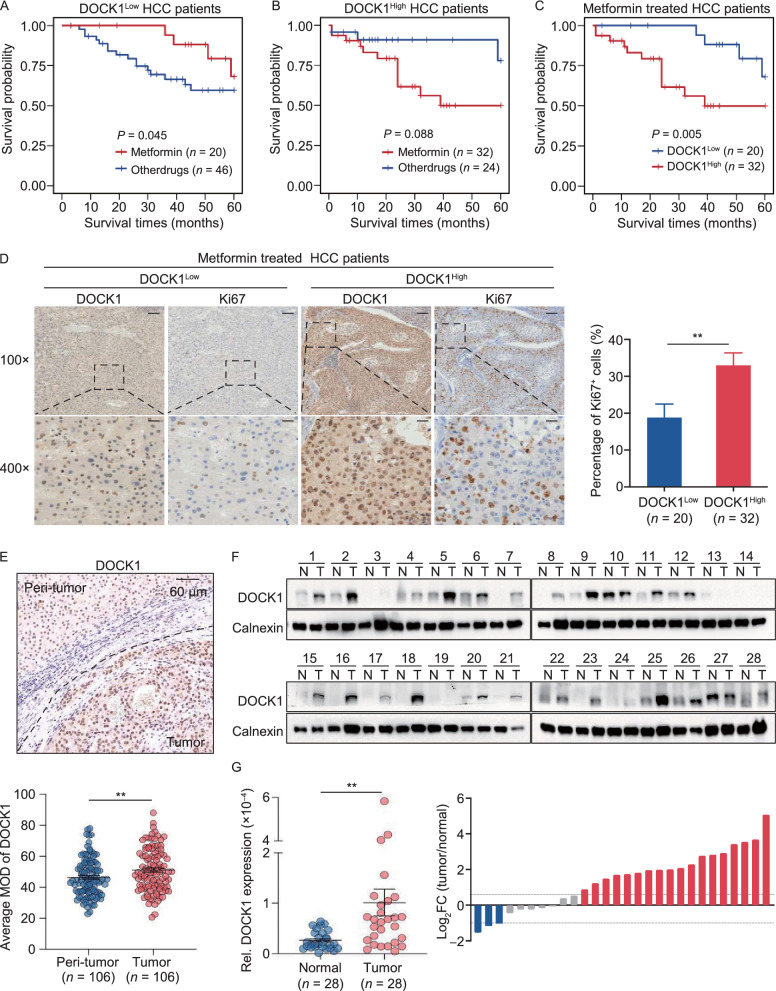
DOCK1 levels determine the anti-tumor activity of metformin in liver cancer patients. (A and B) Kaplan-Meier curves with univariate analyses for patients with metformin treatment versus other drugs treatment in DOCK1^Low^ (A) or DOCK1^High^ (B) diabetic HCC cohort. *P* values were calculated by Breslow (generalized Wilcoxon) test. (C) Kaplan-Meier curves with univariate analyses for DOCK1^Low^ patients versus DOCK1^High^ patients in metformin treated diabetic HCC cohort. *P* values were calculated by Breslow (generalized Wilcoxon) test. (D) Representative images (Left), and quantified data (Right) of Ki67 in metformin used diabetic HCC patients by immunohistochemical staining. Scale bar, 50 µm. (E) Representative images (Top), and quantified data (Bottom) of DOCK1 in HCC patients by immunohistochemical staining. (F) and (G) Western blot and qPCR analysis of DOCK1 expression in 28 pairs of clinically matched tumor-adjacent noncancerous liver tissues (N) and HCC tissues (T). Calnexin served as a loading control. Data are presented as mean ± SEM

Finally, we sought to determine whether DOCK1 expression levels could serve as a potential biomarker for evaluating the therapeutic effectiveness of metformin in liver cancer patients by retrospective evaluation of 122 clinical HCC patients with diabetes. These patients were classified into a metformin-use group and other anti-diabetic drug-use group. Kaplan–Meier analysis showed that there was no difference in overall survival between metformin-use diabetic HCC patients and the other drug-use group (Fig. S5A). Subsequently, we performed immunohistochemical staining for visualization and quantification of DOCK1 expression and classified patients into DOCK1^Low^ (*n* = 66) and DOCK1^High^ (*n* = 56) categories based on mean DOCK1 intensity. Interestingly, metformin appeared to significantly improve the overall survival of the DOCK1^Low^ patients (Fig. 5A), while in contrast, metformin treatment was associated with poor prognosis in DOCK1^High^ patients (Fig. 5B). These results indicated that metformin has apparently inverse therapeutic effects in patients depending on whether DOCK1 levels are relatively low or high. Further analysis of the metformin-use diabetic HCC patients revealed that patients with low DOCK1 levels had better overall survival rates (Fig. 5C). Moreover, Ki67 staining in samples from metformin-treated diabetic HCC patients revealed that DOCK1^Low^ patients exhibited a lower proportion of Ki67-positive cells compared to that in the DOCK1^High^ group (Fig. 5D). Cumulatively, these data demonstrated that DOCK1 levels determine the anti-tumor effectiveness of metformin in HCC patients.

To test this possibility, we next investigated DOCK1 expression levels in HCC patients. Quantitative analysis of DOCK1 immunohistochemistry staining in HCC specimens indicated that DOCK1 was upregulated in tumor tissues compared to that in adjacent normal tissue (Fig. 5E). Consistent with this finding, qPCR and Western blot analyses showed that both RNA and protein levels of DOCK1 were elevated in HCC tissues compared to those in matched adjacent non-cancerous liver tissues (Fig. 5F and 5G). We observed similar results in the GSE124535 RNA-seq dataset generated with 35 pairs of tumor and non-tumor HCC samples (Jiang et al., 2019) (Fig. S5B). Relative expression analysis of DOCK1 in liver cancer samples from The Cancer Genome Atlas (TCGA) showed the same results (Fig. S5C).

The high DOCK1 expression suggests the likelihood of poor metformin performance in HCC patients. It warrants mention that although DOCK1 expression is widely upregulated in HCC patients, tumor heterogeneity still results in differential accumulation of DOCK1 among HCC samples, with some patients exhibiting very low, even undetectable, levels of DOCK1 (Fig. 5F). This finding further suggests that a personalized precision medicine approach should be strongly considered for HCC patients based on their specific DOCK1 levels. Collectively, these data suggest that DOCK1 is upregulated in HCC, and its degree of upregulation can determine the anti-tumor effectiveness of metformin.

## Discussion

**Figure 6:**
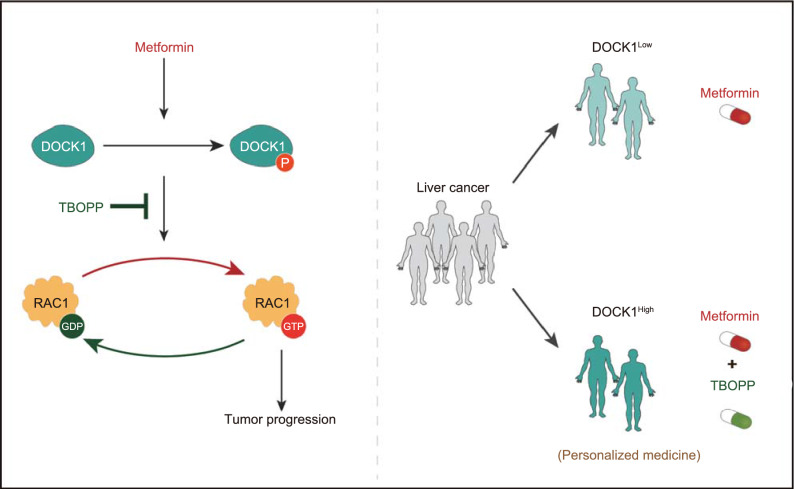
Working model: DOCK1 determines the anti-tumor activity of metformin via DOCK1/RAC1 axis. Schematic showing that metformin promotes DOCK1 phosphorylation, which activates RAC1 to facilitate cell survival, leading to metformin resistance in liver cancer. Therefore, DOCK1^Low^ HCC patients may benefit from metformin monotherapy, while among patients with high DOCK1 expression, combining metformin with TBOPP may provide a promising personalized therapeutic strategy

Metformin is established as a potential anti-tumor agent against various tumors (Pollak 2012; Pernicova and Korbonits 2014). However, some clinical trials have shown poor results, suggesting that metformin may not be suitable for all cancer types due to tumor heterogeneity. Hence, valuable insights towards its clinical application as an anti-tumor agent could be obtained through identification of the subpopulation of cancer patients who are likely to benefit from metformin treatment. Here, we show that metformin selectively improved survival rates among HCC patients with low DOCK1 levels, but not for patients exhibiting high DOCK1 expression (Figs. 5A–C and S5A). Mechanistically, metformin facilitates the activation of RAC1 by phosphorylating DOCK1 (Fig. 3D, 3H and 3J), which in turn attenuates the anti-tumor effects of metformin, leading to the observed unresponsiveness in liver cancer. Combined treatment of metformin with DOCK1-selective inhibitor led to the sensitization of cancer cells to metformin both* in vitro* and* in vivo* (Fig. 4A, 4C, 4D and 4F). Based on these findings, we thus propose the possibility of rational use of metformin in personalized therapeutic strategies against liver cancer that depend on patient levels of DOCK1, with low DOCK1 levels indicating the potential for benefit from metformin administration (Fig. 6).

Numerous genetic alterations have been observed in HCC which lead to dysregulation of TP53, MYC and CTNNB1, for example, thus suggesting these proteins can serve as potential targets for cancer treatment. However, many of these proteins are still difficult to directly target due to their structural characteristics or their essential functions in normal physiological processes (Dang et al. 2017). Here, we found that DOCK1 was significantly upregulated in HCC (Figs. 5E–G and S5B) and that targeting DOCK1 with selective inhibitor resulted in strong synergistic anti-tumor effects when administered with metformin (Fig. 4A, 4C, 4D and 4F). DOCK1 is a canonical GEF which mediates the activation of the small GTPase RAC (Laurin and Côté 2014). In addition to DOCK1, many other GEFs participate in RAC activation (Rossman et al. 2005), and as a result, targeting DOCK1 leads to only modest disruption of RAC physiological function, if at all. Recently, the DOCK1 selective inhibitor TBOPP was identified and reported to suppress cancer cell growth and metastasis *in vivo* (Tajiri et al. 2017). Although DOCK1 selective inhibitors have not yet undergone clinical trials, our study showed that DOCK1-specific inhibitors have strong apparent potential for use as efficient anti-tumor drugs through synergistic enhancement of the anti-tumor effects of metformin. It is also interesting to note that overexpression of RAC1^G12V^ didn’t completely rescue the cells treated with shDOCK1 and metformin, suggesting that DOCK1 might have some other potential functions beyond the DOCK1-RAC1 axis to enhance the cancer cell-killing activity of metformin, which warrants further independent investigation. Moreover, previous studies revealed that DOCK1 promotes tumor proliferation and invasion in glioma and breast cancer (Feng et al. 2011, 2012; Laurin et al. 2013). Here, we found that knockdown of DOCK1 inhibited tumor development in YAP5SA induced orthotopic HCC model (Fig. 2H), suggesting that DOCK1 might directly regulate HCC development.

Liver cancer is highly heterogeneous and traditional two-dimensional cell culture methods have thus far failed to recapitulate the three-dimensional architecture and tumor heterogeneity. Recently a three-dimensional organoid model was developed to overcome these limitations, effectively providing a reliable platform to test patient-specific drug response and for personalizing therapeutic approaches (Bredenoord et al. 2017; Broutier et al. 2017). Here, we established four HCC organoids and confirmed that these patient-derived organoids retained the histological features of the original tumors (Fig. S2A). Our data showed that DOCK1 deficiency resulted in sensitization of the patient-derived HCC organoids to metformin, while metformin combined with TBOPP showed strong, synergistic anti-tumor effects in metformin-resistant organoids (Figs. 2C–D and 4C). Recently, patient-derived organoids were used to accurately predict the response to chemotherapy in metastatic colorectal cancer patients, thus highlighting their clinically informative value. Given that anti-tumor drugs display variable therapeutic effects among different patients, our findings in HCC organoids may guide personalized strategy development for HCC patients.

Moreover, metformin selectively improved the overall survival of HCC patients with low DOCK1 expression in our cohort (Figs. 5A–C and S5A), supporting our conclusion that DOCK1 determines the therapeutic outcome of metformin treatment, and highlighting the clinical significance of our findings. Furthermore, analysis of several datasets showed that DOCK1 is upregulated in liver cancer (Figs. 5E–G, S5B and S5C), which suggested that the combined treatment of metformin with DOCK1 inhibitor could provide clinical benefits for HCC patients who have high levels of DOCK1. In addition, some patients exhibit only low levels of DOCK1, and thus may benefit from metformin monotherapy. Considering the high heterogeneity of DOCK1 expression in HCC patients, personalized therapeutic strategies of metformin may represent the most effective strategy. Clinically, our results suggest the potential implication to determine DOCK1 levels by immunohistochemical analysis of tumor tissues from patient biopsy. Thus, we propose that HCC patients with low DOCK1 levels are recommended to metformin administration, while patients with high DOCK1 levels, which may lead to poor prognosis by activating DOCK1-RAC1 axis, should not be treated with metformin alone. Instead, our study suggests that a combined therapy with DOCK1 selective inhibitors and metformin would be beneficial to these patients. Nevertheless, the specific classification criteria of DOCK1 still need further and larger clinical analysis, so as to find the accurate threshold of DOCK1 expression with the greatest prognostic differences.

Currently, sorafenib and lenvatinib are approved for patients with advanced HCC. While some of these patients exhibited improved clinical outcomes, many other patients cannot tolerate the toxicity of this treatment regimen. As the most frequently prescribed anti-diabetic drug, metformin has a demonstrated record of safety, and therefore the identification of different subtypes of liver cancer patient may provide an expanded range of options for patients with low DOCK1 expression. Future research will verify whether the findings presented here can be extended to a broad range of tumors. It is also worth noting that this study had some limitations. In particular, this retrospective study was based on the cohort recruited from a single center. In addition, the effects of metformin and DOCK1 were not investigated in liver cancer patients without diabetes. Therefore, further clinical trials are needed to evaluate the potential for clinical application.

In conclusion, our study highlights the role of DOCK1 in determining the response of liver cancer cells to metformin treatment and illustrates the inhibition of tumor progression by metformin in liver cancer patients with low DOCK1 expression. Our findings also suggest the potential effectiveness of a metformin-DOCK1 inhibitor combination strategy for treating liver cancer patients with high DOCK1 expression, which warrants further clinical investigation.

## Materials and methods

### Cell lines

HEK293T, PLC and Hep3B cells (From ATCC) were cultured in DMEM (Gibco) supplemented with 10% fetal bovine serum (FBS, Gibco) and 1% penicillin-streptomycin (Hyclone). Huh7 cells (From Stem Cell Bank, Chinese Academy of Science) were cultured in DMEM supplemented with 10% FBS, 2 mmol/L GlutaMAX (Gibco), 1 mmol/L sodium pyruvate (Gibco) and 1% penicillin-streptomycin. SNU423, SNU449 and SNU475 cells (From CoBioer Biosciences) were cultured in RPMI 1640 (Gibco) supplemented with 10% FBS and 1% penicillin-streptomycin. CLC1, CLC11 cells (From Center for Excellence in Molecular Cell Science, Chinese Academy of Science) were cultured in primary medium: RPMI 1640 supplemented with 10% FBS, 1 * ITS (Insulin, Transferrin, Selenium Solution, Gibco) and 40 ng/μL EGF (PeproTech) and 1% penicillin-streptomycin. CLC50 cells (From Center for Excellence in Molecular Cell Science, Chinese Academy of Science) were cultured in primary medium supplemented with 10 μmol/L Y-27632 (Selleck Chemicals), and 5 μmol/L A83-01 (Tocris Bioscience). Cells were incubated at 37 °C with 5% CO_2_.

### CRISPR-Cas9-based screen

Human genome-scale CRISPR knockout library (GeCKO v2) containing 123,411 sgRNAs targeting 19,050 genes was packed into lentiviral particle and transduced into PLC cells at low multiplicity of infection (MOI < 0.3). Following a two-week incubation with or without 1 mmol/L metformin, genomic DNA was extracted and the sgRNA fragment was amplified by PCR. sgRNA abundance was determined by high-throughput sequencing and analyzed by MAGeCKFlute (Wang et al. 2019). Human GeCKO v2 library was a gift from Feng Zhang (Sanjana et al. 2014).

### Tissue specimens

Human HCC and adjacent nontumor liver tissues were collected from patients who underwent surgical resection at The First Affiliated Hospital of USTC (Hefei, China). Informed consent in writing was obtained from each patient and this study was approved by Institutional Research Ethics Committee of The First Affiliated Hospital of USTC. The details of the patient information are shown in Tables S1–4.

### Development of HCC organoids

Organoids were generated according to previously described protocols (Broutier et al. 2017; Nuciforo et al. 2018) with slight modifications. Briefly, tissues (0.25–1 cm^3^) were minced and digested with 2.5 mg/mL collagenase D (Roche), 0.1 mg/mL DNase (Sigma) at 37 °C for 2–3 h. 2,000–5000 cells in 50 µL BME2 (Basement Membrane Extract Type 2, PathClear) were seeded per well in a 24-well plate. Tumoroids-specific isolation medium were added after the BME solidified. Patients information is provided in Table S5

### qPCR

Total RNA was extracted using FastPure Cell/Tissue Total RNA Isolation Kit (Vazyme) and reverse transcription with a HiScript II 1st Strand cDNA Synthesis Kit (Vazyme) following the manufacturer’s instructions. qPCR was performed using SYBR Green Master Mix (Vazyme) on a Bio-Rad CFX Connect Real-Time PCR Detection instrument. Primer sequences are listed in the Table S6. The expression of all samples was normalized to 18S rRNA.

### Western blot analysis

Cells were lysed with RIPA buffer (50 mmol/L Tris-HCl (pH 8.0), 150 mmol/L NaCl, 5 mmol/L EDTA, 0.1% SDS, and 1% NP-40) supplemented with protease inhibitor cocktail, and equal amounts of protein lysate were loaded and separated by SDS-PAGE. Antibodies information are listed in the Table S7.

### Colony formation assays

1,500–5,000 cells were placed in a 6-well plate, and maintained in complete medium supplemented with metformin or TBOPP at indicated dose for approximate 2 weeks. Cells were fixed with ethanol and stained with 0.1% crystal violet.

### HE and immunohistochemistry

Tissues and organoids were fixed in 10% formalin and embedded in paraffin followed by sectioned at 4 µm, then stained with hematoxylin and eosin, or specific antibodies.

### Cell viability analysis

Cell viability was measured using CellTiter-Glo 3D reagent (Promega). A CLARIOstar multimode microplate reader (BMG LABTECH) was used to measure Luminescence.

### RNA sequencing and data analysis

Total RNA was extracted using FastPure Cell/Tissue Total RNA Isolation Kit (Vazyme) following the manufacturer’s instructions, and the RNA integrity was determined by Agilent Bioanalyzer 2100. Libraries were generated using the NEB Next Ultra RNA Library Prep Kit for Illumina (NEB). RNA-seq was performed on Illumina NovaSeq platform by Novogene. Reads were first aligned to the human reference genome hg19 with STAR, transcripts were assembled by StringTie v1.3.4d, and gene expression analysis was performed using DESeq2 v1.28.1. Pathway enrichment analysis were performed by Metascape (Zhou et al. 2019).

### *In vitro* RAC1 activation assay

Activated Rac1 was detected by co-precipitation of active Rac1 with the GST-tagged PBD as described previously (Fukui et al. 2001).

### Immunoprecipitation

Cells were lysed in IP buffer (20 mmol/L HEPES pH 7.5, 150 mmol/L NaCl, 2 mmol/L EDTA, 1% NP40) supplemented with protease inhibitors. 50–100 µg of lysate were saved as input sample for quantitation of total DOCK1. Equivalent protein was immunoprecipitated with anti-Flag antibody for 4–6 h at 4 °C, followed by incubation with protein A/G-Sepharose for 1 h. Beads were washed twice with IP buffer, and boiled in 2× loading buffer. Protein samples were analyzed by SDS-PAGE.

### Plasmids

shRNAs in the PLKO.1 vector against human and mouse DOCK1 were commercially purchased (Sigma-Aldrich). shRNA targeting the 3′UTR of DOCK1 were constructed in the PLKO.1 vector, the target sequences are listed in Table S8. The coding sequences of human DOCK1, DOCK1^△DHR2^ and DOCK1^Y722F/Y1811F^ were constructed in the pCDH-3×flag vector. The coding sequences of RAC1, and RAC1^G12V^ were constructed in the pSIN-3×flag vector. The Act-PB transposase, PB[Act-RFP]DS and PB[CMV-myc-YAP-5SA]DS plasmids were a gift from Prof. Bin Zhao. To make the PB[CMV-myc-YAP-5SA-U6-shNTC]DS and PB[CMV-myc-YAP-5SA-U6-shDOCK1]DS plasmids, two U6-shRNA fragments targeting mouse DOCK1 or NTC were ligated with the SwaI digested PB[CMV-myc-YAP-5SA]DS plasmid using Gibson assembly. The pT/Caggs-NRASV12, PT2/C-Luc/PGK-SB13, and pT2/shp53/GFP4 were gifts from John Ohlfest (Addgene).

### Animal experiments

All animal studies were conducted with approval from the Animal Research Ethics Committee of University of Science and Technology of China. Mice were purchased from Beijing Vital River Laboratory Animal Technology Co., Ltd.

For the* in vivo* xenograft experiment, 8 × 10^6^ PLC cells were subcutaneously injected into 5-week old male BALB/c nude mice followed with the indicated treatment. Tumor-bearing mice were randomized into different groups at the time of treatment initiation. Tumors were measured by caliper every 3 day, and tumor volumes were calculated using the following formula: Tumor volume (mm^3^) = length (mm) × width (mm) × depth (mm) × 0.52.

For the orthotopic liver cancers model, indicated plasmids suspending in normal saline solution were delivered to 8-week old (for NRAS^G12V^/shP53 induced liver cancer) or 6-week old (for YAP5SA induced liver cancer) C57BL/6 mice by hydrodynamic tail vein injection (HDI). Mice were sacrificed approximate 2 or 4 months after plasmids injection, respectively.

Metformin (MedChemExpress) was provided in drinking water when tumor reached 100 mm^3^ in volume (xenograft) or 3 (for NRAS^G12V^/shP53 induced liver cancer) or 4 weeks (for YAP5SA induced liver cancer) after plasmids injection. TBOPP (Topscience) was dissolved in PBS containing 10% DMSO and 12.5% Cremophol EL and injected by the tail vein at the indicated time.

### Statistical analysis

Data are presented as the mean ± SD or mean ± SEM of at least three independent experiments as indicated. Student’s *t* test was used to compare two groups. ANOVA was used for multiple comparisons (GraphPad Software). *P < 0.05; **P < 0.01; ***P < 0.001; ns: not significant.

## Supplementary Information

Below is the link to the electronic supplementary material.

Supplementary file1 (PDF 1526 kb)

## Data Availability

The RNA-seq dataset produced in this study are available in the Gene Expression Omnibus: GSE181521 (https://www.ncbi.nlm.nih.gov/geo/query/acc.cgi?acc=GSE181521).

## Abbreviations

DHR2: DOCK homology region-2
DOCK1: dedicator of cytokinesis 1
GeCKO v2: genome-scale CRISPR knock-Out library
GEF: guanine nucleotide exchange factor
GO: Gene Ontology
HCC: hepatocellular carcinoma
IC_50_: half-maximal inhibitory concentrations
NTC: non-targeting control
PLC: PLC/PRF/5
qPCR: quantitative real-time polymerase chain reaction
RAC1: small GTPase Ras-related C3 botulinum toxin substrate 1
TBOPP: 1-(2-(30-(Trifluoromethyl)-[1,10-biphenyl]-4-yl)-2-oxoethyl)-5-pyrrolidinylsulfonyl-2(1H)-pyridone
TCGA: The Cancer Genome Atlas

## References

[CR47] Ally Adrian, Balasundaram Miruna, Carlsen Rebecca, Chuah Eric, Clarke Amanda, Dhalla Noreen, Holt Robert A., Jones Steven J.M., Lee Darlene, Ma Yussanne (2017). Comprehensive and integrative genomic characterization of hepatocellular carcinoma. Cell.

[CR1] Ben Sahra I, Regazzetti C, Robert G, Laurent K, Le Marchand-Brustel Y, Auberger P, Tanti J-F, Giorgetti-Peraldi S, Bost F (2011). Metformin, independent of AMPK, induces mTOR inhibition and cell-cycle arrest through REDD1. Can Res.

[CR2] Bonnefont-Rousselot D, Raji B, Walrand S, Gardès-Albert M, Jore D, Legrand A, Peynet J, Vasson MP (2003). An intracellular modulation of free radical production could contribute to the beneficial effects of metformin towards oxidative stress. Metabolism.

[CR3] Bray F, Ferlay J, Soerjomataram I, Siegel RL, Torre LA, Jemal A (2018). Global cancer statistics 2018: GLOBOCAN estimates of incidence and mortality worldwide for 36 cancers in 185 countries. CA Cancer J Clin.

[CR4] Bredenoord AL, Clevers H, Knoblich JA (2017) Human tissues in a dish: The research and ethical implications of organoid technology. *Science (New York, NY)* 355 10.1126/science.aaf9414 28104841

[CR5] Broutier L, Mastrogiovanni G, Verstegen MM, Francies HE, Gavarró LM, Bradshaw CR, Allen GE, Arnes-Benito R, Sidorova O, Gaspersz MP (2017). Human primary liver cancer-derived organoid cultures for disease modeling and drug screening. Nat Med.

[CR6] Brugnera E, Haney L, Grimsley C, Lu M, Walk SF, Tosello-Trampont A-C, Macara IG, Madhani H, Fink GR, Ravichandran KS (2002). Unconventional Rac-GEF activity is mediated through the Dock180-ELMO complex. Nat Cell Biol.

[CR7] Carlson CM, Frandsen JL, Kirchhof N, McIvor RS, Largaespada DA (2005). Somatic integration of an oncogene-harboring Sleeping Beauty transposon models liver tumor development in the mouse. Proc Natl Acad Sci USA.

[CR8] Chen H-P, Shieh J-J, Chang C-C, Chen T-T, Lin J-T, Wu M-S, Lin J-H, Wu C-Y (2013). Metformin decreases hepatocellular carcinoma risk in a dose-dependent manner: population-based and in vitro studies. Gut.

[CR10] Côté J-F, Vuori K (2002). Identification of an evolutionarily conserved superfamily of DOCK180-related proteins with guanine nucleotide exchange activity. J Cell Sci.

[CR11] Dang CV, Reddy EP, Shokat KM, Soucek L (2017). Drugging the 'undruggable' cancer targets. Nat Rev Cancer.

[CR12] Feng H, Hu B, Liu K-W, Li Y, Lu X, Cheng T, Yiin J-J, Lu S, Keezer S, Fenton T (2011). Activation of Rac1 by Src-dependent phosphorylation of Dock 180(Y1811) mediates PDGFRα-stimulated glioma tumorigenesis in mice and humans. J Clin Invest.

[CR13] Feng H, Hu B, Jarzynka MJ, Li Y, Keezer S, Johns TG, Tang CK, Hamilton RL, Vuori K, Nishikawa R (2012). Phosphorylation of dedicator of cytokinesis 1 (Dock180) at tyrosine residue Y722 by Src family kinases mediates EGFRvIII-driven glioblastoma tumorigenesis. Proc Natl Acad Sci USA.

[CR14] Fukui Y, Hashimoto O, Sanui T, Oono T, Koga H, Abe M, Inayoshi A, Noda M, Oike M, Shirai T, Sasazuki T (2001). Haematopoietic cell-specific CDM family protein DOCK2 is essential for lymphocyte migration. Nature.

[CR15] Galdieri L, Gatla H, Vancurova I, Vancura A (2016). Activation of AMP-activated protein kinase by metformin induces protein acetylation in prostate and ovarian cancer cells. J Biol Chem.

[CR16] Guo X, Zhao Y, Yan H, Yang Y, Shen S, Dai X, Ji X, Ji F, Gong X-G, Li L (2017). Single tumor-initiating cells evade immune clearance by recruiting type II macrophages. Genes Dev.

[CR17] Gwinn DM, Shackelford DB, Egan DF, Mihaylova MM, Mery A, Vasquez DS, Turk BE, Shaw RJ (2008). AMPK phosphorylation of raptor mediates a metabolic checkpoint. Mol Cell.

[CR18] Inoki K, Zhu T, Guan K-L (2003). TSC2 mediates cellular energy response to control cell growth and survival. Cell.

[CR19] Jiang Y, Sun A, Zhao Y, Ying W, Sun H, Yang X, Xing B, Sun W, Ren L, Hu B (2019). Proteomics identifies new therapeutic targets of early-stage hepatocellular carcinoma. Nature.

[CR20] Jin H, Shi Y, Lv Y, Yuan S, Ramirez CFA, Lieftink C, Wang L, Wang S, Wang C, Dias MH (2021). EGFR activation limits the response of liver cancer to lenvatinib. Nature.

[CR21] Kiyokawa E, Hashimoto Y, Kobayashi S, Sugimura H, Kurata T, Matsuda M (1998). Activation of Rac1 by a Crk SH3-binding protein, DOCK180. Genes Dev.

[CR22] Kudo M, Finn RS, Qin S, Han K-H, Ikeda K, Piscaglia F, Baron A, Park J-W, Han G, Jassem J (2018). Lenvatinib versus sorafenib in first-line treatment of patients with unresectable hepatocellular carcinoma: a randomised phase 3 non-inferiority trial. The Lancet.

[CR23] Laurin M, Côté J-F (2014). Insights into the biological functions of Dock family guanine nucleotide exchange factors. Genes Dev.

[CR24] Laurin M, Huber J, Pelletier A, Houalla T, Park M, Fukui Y, Haibe-Kains B, Muller WJ, Côté J-F (2013). Rac-specific guanine nucleotide exchange factor DOCK1 is a critical regulator of HER2-mediated breast cancer metastasis. Proc Natl Acad Sci USA.

[CR25] Lee J, Yesilkanal AE, Wynne JP, Frankenberger C, Liu J, Yan J, Elbaz M, Rabe DC, Rustandy FD, Tiwari P (2019). Effective breast cancer combination therapy targeting BACH1 and mitochondrial metabolism. Nature.

[CR26] Li Y, Xu S, Mihaylova MM, Zheng B, Hou X, Jiang B, Park O, Luo Z, Lefai E, Shyy JYJ (2011). AMPK phosphorylates and inhibits SREBP activity to attenuate hepatic steatosis and atherosclerosis in diet-induced insulin-resistant mice. Cell Metab.

[CR27] Llovet JM, Ricci S, Mazzaferro V, Hilgard P, Gane E, Blanc J-F, de Oliveira AC, Santoro A, Raoul J-L, Forner A (2008). Sorafenib in advanced hepatocellular carcinoma. N Engl J Med.

[CR28] Lord SR, Cheng W-C, Liu D, Gaude E, Haider S, Metcalf T, Patel N, Teoh EJ, Gleeson F, Bradley K (2018). Integrated pharmacodynamic analysis identifies two metabolic adaption pathways to metformin in breast cancer. Cell Metab.

[CR29] Nathan DM, Buse JB, Davidson MB, Ferrannini E, Holman RR, Sherwin R, Zinman B (2009). Medical management of hyperglycemia in type 2 diabetes: a consensus algorithm for the initiation and adjustment of therapy: a consensus statement of the American diabetes association and the european association for the study of diabetes. Diabetes Care.

[CR30] Nuciforo S, Fofana I, Matter MS, Blumer T, Calabrese D, Boldanova T, Piscuoglio S, Wieland S, Ringnalda F, Schwank G (2018). Organoid models of human liver cancers derived from tumor needle biopsies. Cell Rep.

[CR31] Pernicova I, Korbonits M (2014). Metformin–mode of action and clinical implications for diabetes and cancer. Nat Rev Endocrinol.

[CR32] Pollak MN (2012). Investigating metformin for cancer prevention and treatment: the end of the beginning. Cancer Discov.

[CR33] Qiu Z, Li H, Zhang Z, Zhu Z, He S, Wang X, Wang P, Qin J, Zhuang L, Wang W (2019). A Pharmacogenomic Landscape in Human Liver Cancers. Cancer Cell.

[CR34] Quinn BJ, Kitagawa H, Memmott RM, Gills JJ, Dennis PA (2013). Repositioning metformin for cancer prevention and treatment. Trends Endocrinol Metab.

[CR35] Rossman KL, Der CJ, Sondek J (2005). GEF means go: turning on RHO GTPases with guanine nucleotide-exchange factors. Nat Rev Mol Cell Biol.

[CR36] Sanjana NE, Shalem O, Zhang F (2014). Improved vectors and genome-wide libraries for CRISPR screening. Nat Methods.

[CR37] Sun X, Niu X, Chen R, He W, Chen D, Kang R, Tang D (2016). Metallothionein-1G facilitates sorafenib resistance through inhibition of ferroptosis. Hepatology.

[CR38] Tajiri H, Uruno T, Shirai T, Takaya D, Matsunaga S, Setoyama D, Watanabe M, Kukimoto-Niino M, Oisaki K, Ushijima M (2017). Targeting ras-driven cancer cell survival and invasion through selective inhibition of DOCK1. Cell Rep.

[CR39] Vancura A, Bu P, Bhagwat M, Zeng J, Vancurova I (2018). Metformin as an anticancer agent. Trends Pharmacol Sci.

[CR40] Wang B, Wang M, Zhang W, Xiao T, Chen CH, Wu A, Wu F, Traugh N, Wang X, Li Z (2019). Integrative analysis of pooled CRISPR genetic screens using MAGeCKFlute. Nat Protoc.

[CR41] Wei L, Lee D, Law C-T, Zhang MS, Shen J, Chin DW-C, Zhang A, Tsang FH-C, Wong CL-S, Ng IO-L (2019). Genome-wide CRISPR/Cas9 library screening identified PHGDH as a critical driver for Sorafenib resistance in HCC. Nat Commun.

[CR42] Wiesner SM, Decker SA, Larson JD, Ericson K, Forster C, Gallardo JL, Long C, Demorest ZL, Zamora EA, Low WC (2009). De novo induction of genetically engineered brain tumors in mice using plasmid DNA. Can Res.

[CR43] Xie J, Xia L, Xiang W, He W, Yin H, Wang F, Gao T, Qi W, Yang Z, Yang X (2020). Metformin selectively inhibits metastatic colorectal cancer with the KRAS mutation by intracellular accumulation through silencing MATE1. Proc Natl Acad Sci U S A.

[CR44] Xu X, Barry DC, Settleman J, Schwartz MA, Bokoch GM (1994). Differing structural requirements for GTPase-activating protein responsiveness and NADPH oxidase activation by Rac. J Biol Chem.

[CR45] Zhou G, Myers R, Li Y, Chen Y, Shen X, Fenyk-Melody J, Wu M, Ventre J, Doebber T, Fujii N (2001). Role of AMP-activated protein kinase in mechanism of metformin action. J Clin Invest.

[CR46] Zhou Y, Zhou B, Pache L, Chang M, Khodabakhshi AH, Tanaseichuk O, Benner C, Chanda SK (2019). Metascape provides a biologist-oriented resource for the analysis of systems-level datasets. Nat Commun.

