# Correction to: Genome-wide CRISPR screen identifies synthetic lethality between DOCK1 inhibition and metformin in liver cancer

**DOI:** 10.1093/procel/pwaf036

**Published:** 2025-06-04

This is a correction to: Junru Feng, Hui Lu, Wenhao Ma, Wenjing Tian, Zhuan Lu, Hongying Yang, Yongping Cai, Pengfei Cai, Yuchen Sun, Zilong Zhou, Jiaqian Feng, Jiazhong Deng, Ying Shu, Kun Qu, Weidong Jia, Ping Gao, Huafeng Zhang, Genome-wide CRISPR screen identifies synthetic lethality between DOCK1 inhibition and metformin in liver cancer, Protein & Cell, Volume 13, Issue 11, November 2022, Pages 825–841, https://doi.org/10.1007/s13238-022-00906-6

The *Protein & Cell* routine checking system detected a duplication in images within Fig. 4C (left panel). After a thorough review of the data files submitted, the authors found that in both the initially submitted version and the revised version, the data were correct. After the manuscript was accepted, in the process of re-formatting the figures, the same image was mistakenly used twice, resulting in an error in the final published version. The corrected figure is shown below.


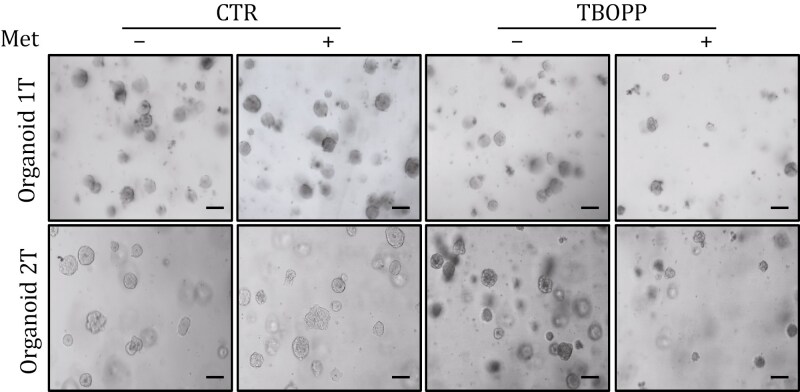


These details have been corrected only in this correction notice to preserve the published version of record.

