# Supplementary material for: Genome-wide CRISPR screen identifies synthetic lethality between DOCK1 inhibition and metformin in liver cancer
Source: Protein Cell. 2022 Feb 26;13(11):825–41. doi: 10.1007/s13238-022-00906-6 (PMC9237198; doi:10.1007/s13238-022-00906-6)
Supplement: Supplementary file 1 — Supplementary file1 (PDF 1526 kb) [file 13238_2022_906_MOESM1_ESM.pdf]

Supplementary Information

Supplementary Figure legends

Supplementary Fig 1. CRISPR-Cas9 library screening identifies DOCK1 as a determinant for metformin sensitivity

(A) Normalized beta-score distribution in control and metformin-treated group.

(B) List of top 15 ranked genes in Fig. 1C.

(C) Pairwise correlation of gene expression and IC_{50} or AUC. *R* is Pearson correlation coefficient.

(D) Colony formation assay of nine liver cancer cell lines treated with 0 mM, 1 mM, 2 mM, 4 mM, and 8 mM metformin for approximately two weeks.

(E) The individual sgRNA counts of DOCK1 in control and metformin-treated group.

(F) IC_{50} curves of metformin in PLC cells stably expressing shDOCK1 or NTC.

(G) Colony formation assay were performed in the indicated SNU449 cell lines with or without 2 mM metformin treatment (Top left). Cell number were quantified (Top right). DOCK1 levels were determined by western blot (Bottom).

Data are presented as mean ± SD.

Supplementary Fig 2. Inhibition of DOCK1 sensitizes liver cancer cells to metformin *in vivo* and *in vitro*

(A) Representative images of HE staining in HCC tissues and organoids. Scale bar, 50 μm.

(B) Organoid 2T stably expressing NTC or shDOCK1 were treated with or without metformin (2.5mM) then the Ki67 level was analyzed by immunofluorescence. Nucleus was

stained by DAPI. Scale bar, 50 μm.

(C) Representative images (Left) and quantified data (Right) of Ki67 in tumor tissues described in Fig. 2H. Scale bar, 50 μm.

(D) Tumor images (Left) and mass (Right) of each group described in Fig. 2J.

Data are presented as mean ± SEM.

Supplementary Fig 3. RAC1 activation contributes to DOCK1-mediated cancer cell insensitivity to metformin

(A) Western blot analysis of DOCK1, RAC1 expression and RAC1-GTP level in the indicated PLC cell lines.

(B) Colony formation assay were performed in the indicated PLC cell lines with or without 1mM metformin treatment (Left). Cell number were quantified (Middle). The expression of DOCK1 was detected by western blot (Right).

(C) Colony formation assay in the indicated PLC cell lines with or without 1mM metformin treatment (Left). Cell number were quantified (Right).

(D) qPCR analysis of DOCK1 expression in PLC cells treated with 0 mM, 1 mM, 2 mM, and 5 mM metformin for 48 hrs.

(E) 293T cells stably expressing Flag-DOCK1, Flag-DOCK1$^{Y722/1811F}$ were treated with or without 5 mM metformin for 48 hrs, followed by immunoprecipitation using anti-Flag antibody. pan-phospho-tyrosine were analyzed by western blot.

(F) Western blot analysis of DOCK1 expression in PLC cells stably expressing shRNA targeting DOCK1 and further infected with EV or DOCK1$^{Y722/1811F}$ lentivirus.

Data are presented as mean ± SD.

Supplementary Fig 4. Synergistic effects of TBOPP and metformin both *in vivo* and *in vitro*

(A) Western blot analysis of DOCK1, RAC1 expression and RAC1-GTP level in PLC cells treated with 0 μM, 0.75 μM, 1 μM, 2 μM TBOPP for 48 hrs.

(B) Colony formation assay of PLC cells treated with increasing concentrations of TBOPP for approximately two weeks.

(C) Synergistic anti-tumor effect between TBOPP and metformin (2mM) in SNU449 (Left) and Hep3B (Right) cells. Cell number were quantified.

(D) IC_{50} curves of TBOPP in organoid 1T and 2T.

(E-G) PLC xenografts were treated with 8 mg/kg, 16 mg/kg TBOPP (i.v. twice a week) (n = 5 for each group). Tumor sizes (E) and body weight (F) were measured every 3 days. Tumor images (G) were collected at the end of the experiment.

(H) Western blot analysis of RAC1 expression and RAC1-GTP level in tumors of each group in Supplementary Fig. 4G.

(I) Body weight of mice in Fig. 4D were collected every 3 days.

Data are presented as mean ± SD in (D). For (E), (F) and (I), data are presented as mean ± SEM.

Supplementary Fig 5. DOCK1 levels determine the anti-tumor activity of metformin in liver cancer patients

(A) Kaplan-Meier curves with univariate analyses for patients with metformin treatment

versus other-drug treatment in diabetic HCC cohort. *P* values were calculated by Breslow (generalized Wilcoxon) test.

(B) DOCK1 expression in 35 pairs of clinically matched tumor-adjacent noncancerous liver tissues (Normal) and HCC tissues (Tumor) in RNA-seq dataset GSE124535.

(C) DOCK1 expression in normal liver (Normal) and HCC (Tumor) in the TCGA project.

Data are presented as mean ± SEM.

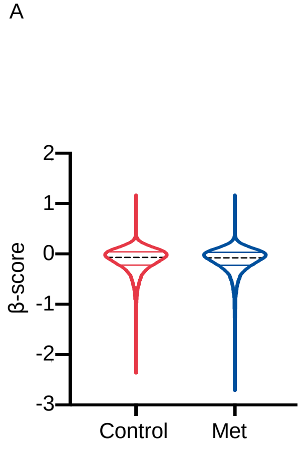

B

Rank	Gene	Differential β-score
1	hsa-mir-548i-3	-0.69
2	UGT2A2	-0.66
3	POTEJ	-0.64
4	TMED7-TICAM2	-0.63
5	hsa-mir-6893	-0.62
6	hsa-mir-6886	-0.61
7	CMIP	-0.58
8	KRTAP2-4	-0.55
9	ARPP19	-0.53
**10**	**DOCK1**	**-0.53**
11	TSPAN11	-0.51
12	TMEM143	-0.50
13	MESDC2	-0.50
14	ZCCHC24	-0.48
15	MCTS1	-0.47

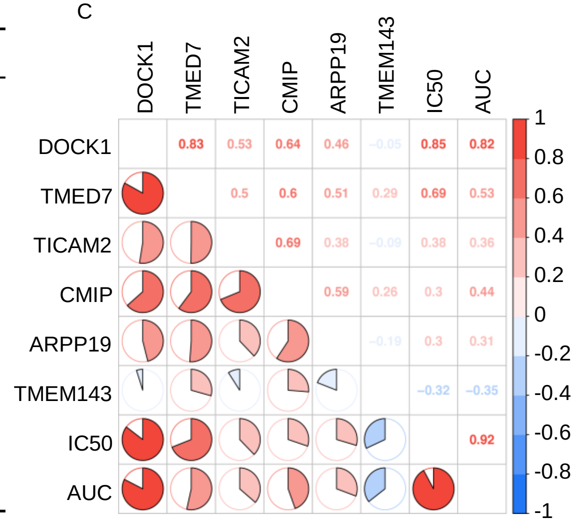

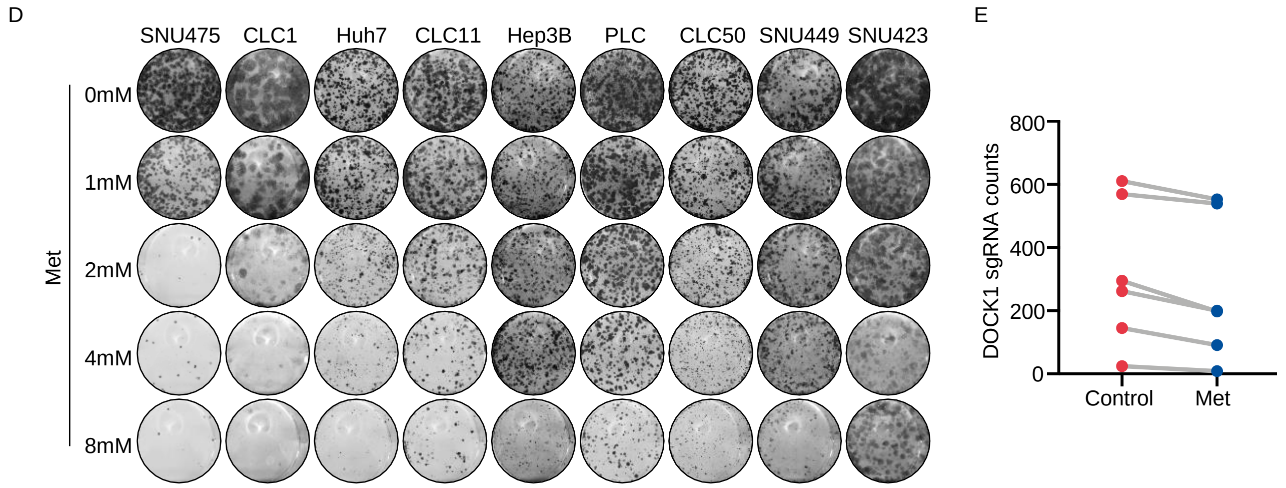

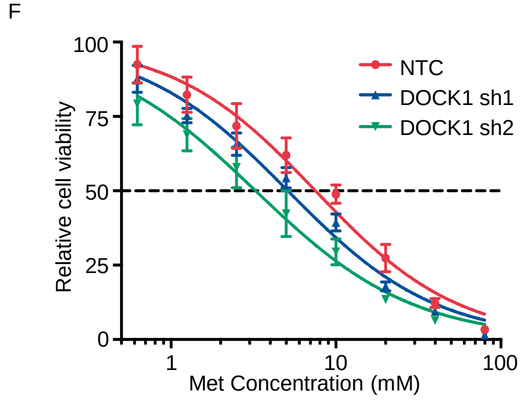

	NTC	DOCK1 sh1	DOCK1 sh2
IC50(mM)	7.52	5.11	3.26

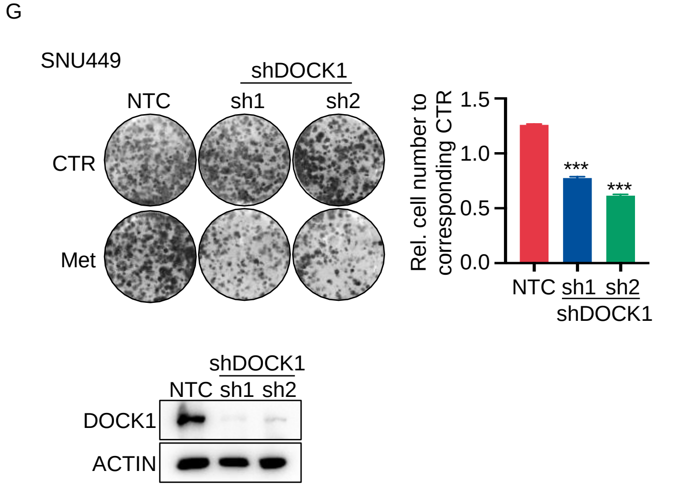

supFig1. CRISPR-Cas9 library screening identifies DOCK1 as a determinant for metformin sensitivity

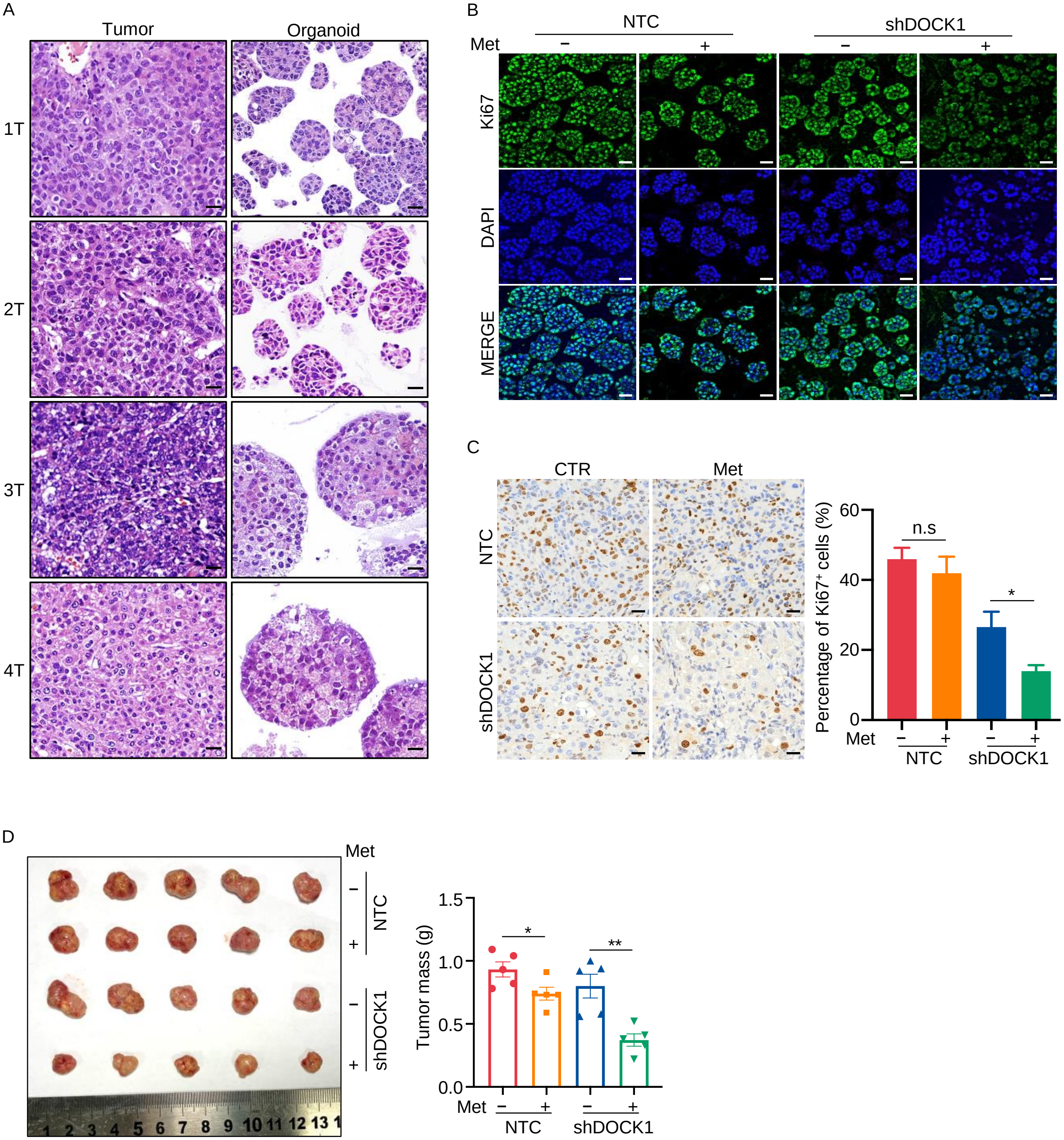

supFig2. Inhibition of DOCK1 sensitizes liver cancer cells to metformin *in vivo* and *in vitro*

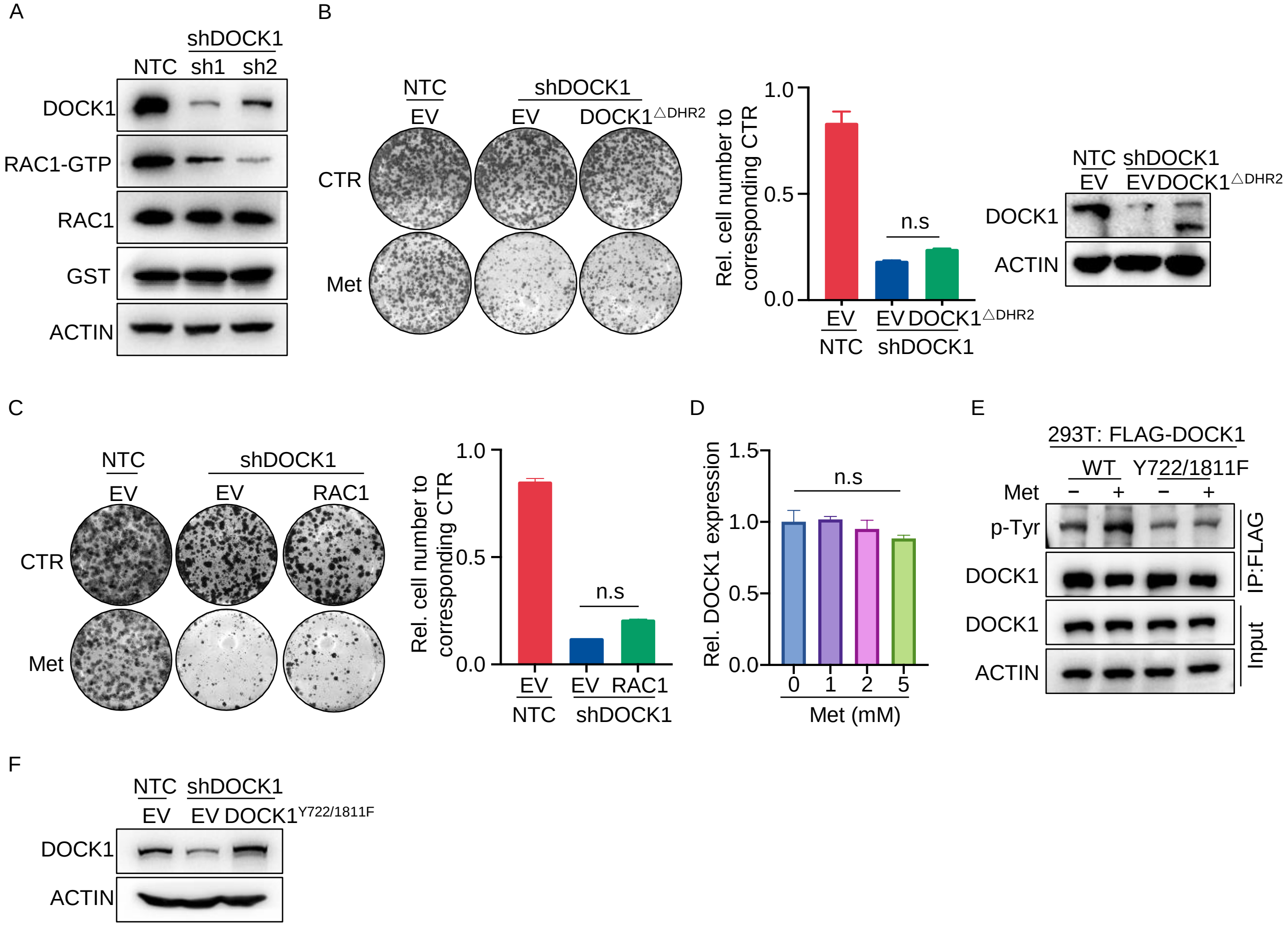

supFig3. RAC1 activation contributes to DOCK1-mediated cancer cell insensitivity to metformin

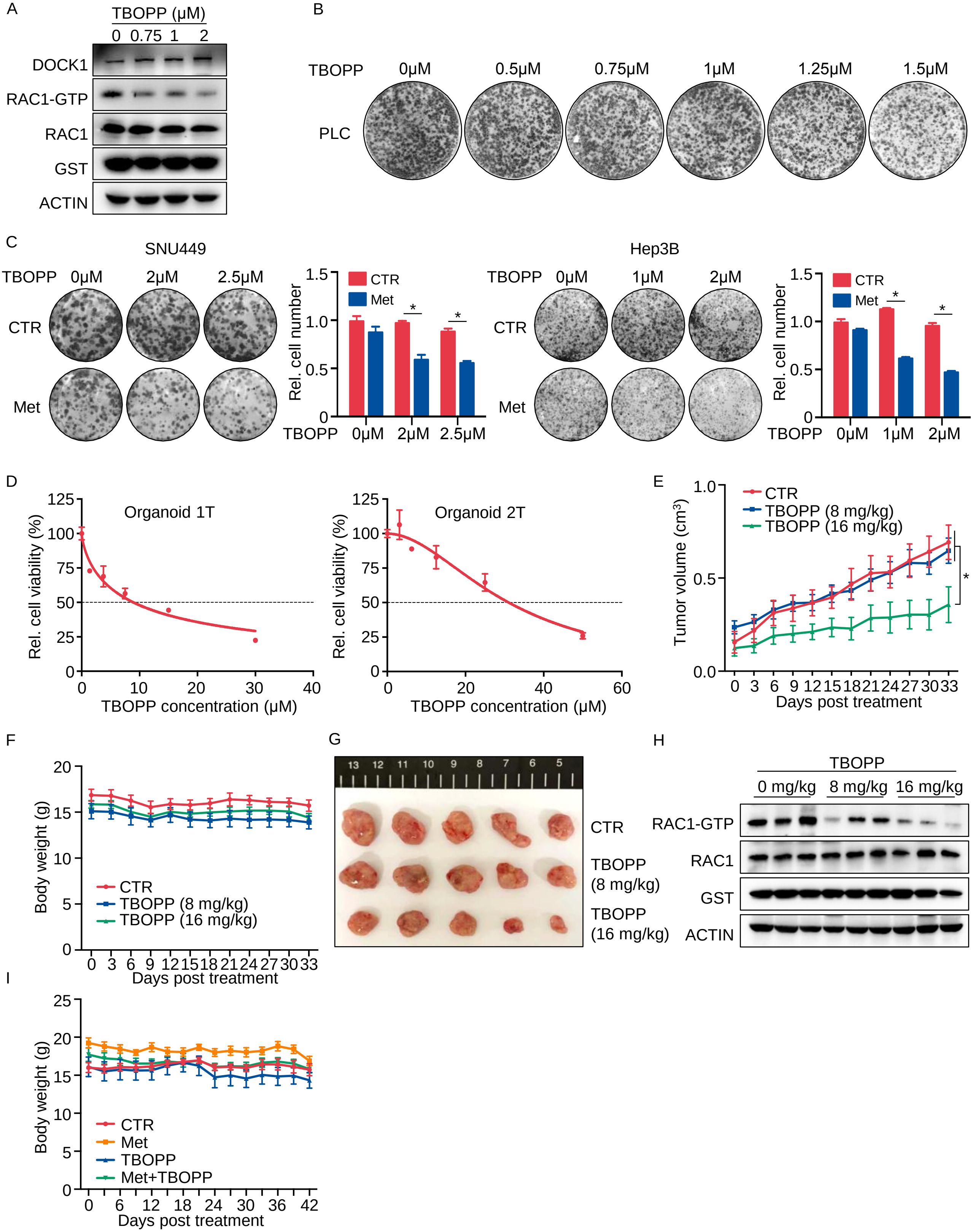

supFig4. Synergistic effects of TBOPP and metformin both *in vivo* and *in vitro*

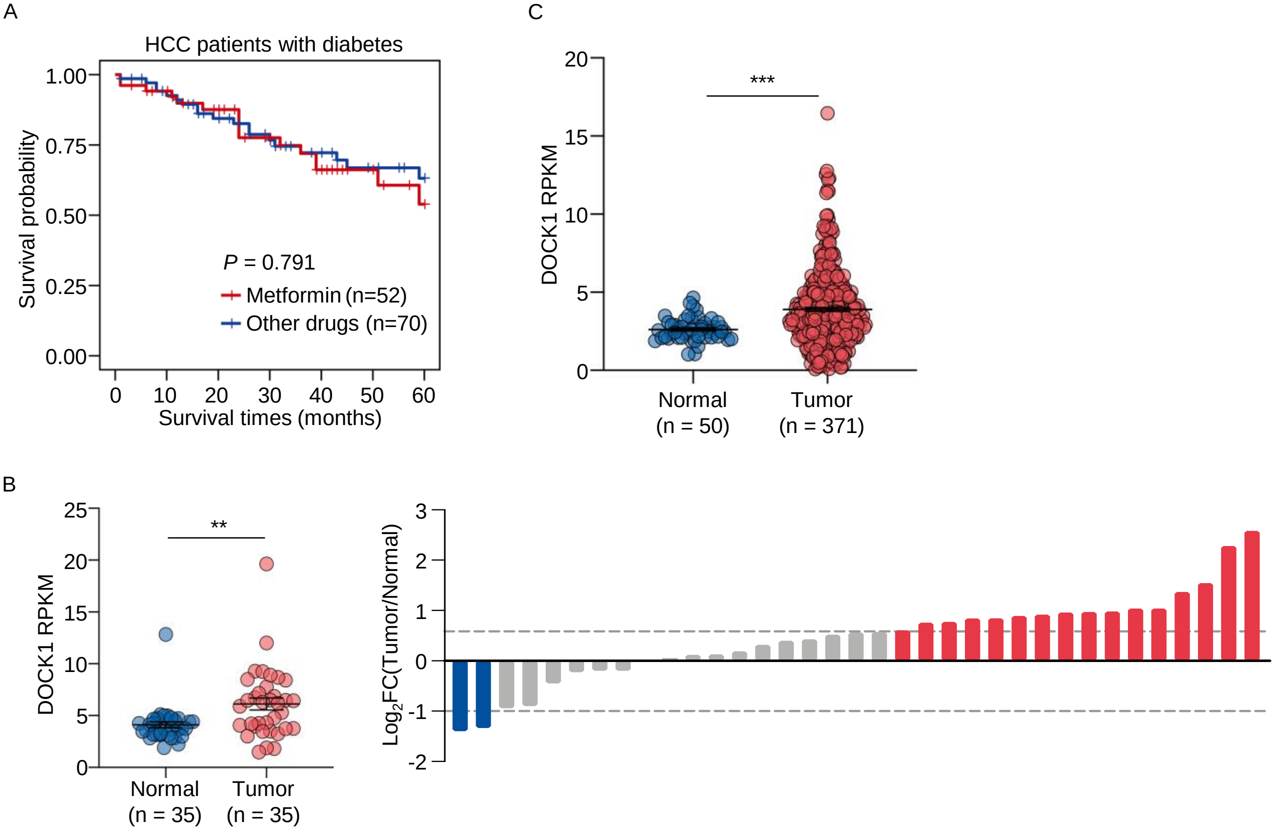

supFig5. DOCK1 levels determine the anti-tumor activity of metformin in liver cancer patients

Supplementary Table 1. Clinicopathological characteristics of HCC patients with T2DM, according to hypoglycemic drugs use. Related to Fig. S5A

Characteristics	Total	Hypoglycemic drugs use		Chi-square
	No. (%)	Metformin No. (%)	Other-drugs No. (%)	*P* value
**Gender**				0.3912
Male	96 (78.69)	39 (31.97)	57 (46.72)	
Female	26 (21.31)	13 (10.66)	13 (10.66)	
**Age at diagnosis, years**				0.0666
< 60	34 (27.87)	10 (27.87)	24 (19.67)	
≥ 60	88 (72.13)	42 (72.13)	46 (37.70)	
**HBV**				0.4759
Negative	38 (31.15)	18 (14.75)	20 (16.39)	
Positive	84 (68.85)	34 (27.87)	50 (40.98)	
**HCV**				0.2962
Negative	117 (95.9)	51 (41.8)	66 (54.10)	
Positive	5 (4.10)	1 (0.82)	4 (3.28)	
**AFP (ng/mL)**				0.4738
< 400	94 (77.05)	38 (31.15)	56 (45.9)	
≥ 400	27 (22.13)	13 (10.66)	14 (11.48)	
Unknown	1 (0.819)	1 (0.819)	0 (0.00)	
**Cirrhosis**				0.152
Absent	34 (27.87)	18 (14.75)	16 (13.11)	
Present	88 (72.13)	34 (27.87)	54 (44.26)	
**Clinical stage (BCLC)**				0.1782
A	55 (45.08)	28 (22.95)	27 (22.13)	
B	12 (9.84)	3 (2.46)	9 (7.38)	
C	55 (45.08)	21 (17.21)	34 (27.87)	
**Pathological grading**				0.667
Well differentiated	20 (16.39)	7 (5.74)	13 (10.66)	
Moderately differentiated	78 (63.93)	36 (29.51)	42 (34.43)	
Poorly & un-differentiated	14 (11.48)	6 (4.92)	8 (6.56)	
Unknown	10 (8.20)	3 (2.46)	7 (5.74)	

Supplementary Table 2. Clinicopathological characteristics of DOCK1^{Low} HCC patients with T2DM, according to hypoglycemic drugs use. Related to Fig. 5A

Characteristics	Total No. (%)	Hypoglycemic drugs use		Chi-square
		Metformin No. (%)	Other-drugs No. (%)	*P* value
**Gender**				0.6318
Male	55 (83.33)	16 (24.24)	39 (59.09)	
Female	11 (16.67)	4 (6.06)	7 (10.61)	
**Age at diagnosis, years**				0.0478
< 60	13 (19.70)	1 (1.52)	12 (18.18)	
≥ 60	53 (80.30)	19 (28.79)	34 (51.52)	
**HBV**				0.9718
Negative	20 (30.30)	6 (9.09)	14 (21.21)	
Positive	46 (69.7)	14 (21.21)	32 (48.48)	
**HCV**				0.8118
Negative	62 (93.94)	19 (28.79)	43 (65.15)	
Positive	4 (6.06)	1 (1.52)	3 (4.55)	
**AFP (ng/mL)**				0.1652
< 400	53 (80.30)	14 (21.21)	39 (59.09)	
≥ 400	13 (19.70)	6 (9.09)	7 (10.61)	
**Cirrhosis**				0.6540
Absent	19 (28.79)	5 (7.58)	14 (21.21)	
Present	47 (71.21)	15 (22.73)	32 (48.48)	
**Clinical stage (BCLC)**				0.2890
A	31 (46.97)	12 (18.18)	19 (28.79)	
B	8 (12.12)	1 (1.52)	7 (10.61)	
C	27 (40.91)	7 (10.61)	20 (30.30)	
**Pathological grading**				0.7087
Well differentiated	42 (63.64)	12 (18.18)	30 (45.45)	
Moderately differentiated	8 (12.12)	2 (3.03)	6 (9.09)	
Poorly & un-differentiated	7 (10.61)	3 (4.55)	4 (6.06)	
Unknown	9 (13.64)	3 (4.55)	6 (9.09)	

Supplementary Table 3. Clinicopathological characteristics of DOCK1^{High} HCC patients with T2DM, according to hypoglycemic drugs use. Related to Fig. 5B

Characteristics	Total	Hypoglycemic drugs use		Chi-square
	No. (%)	Metformin No. (%)	Other-drugs No. (%)	*P* value
**Gender**				0.0005
Male	41 (73.21)	23 (41.07)	18 (32.14)	
Female	15 (26.79)	9 (16.07)	6 (10.71)	
**Age at diagnosis, years**				0.0943
< 60	21 (37.50)	9 (16.07)	12 (21.43)	
≥ 60	35 (62.50)	23 (41.07)	12 (21.43)	
**HBV**				0.3216
Negative	18 (32.14)	12 (21.43)	6 (10.71)	
Positive	38 (67.86)	20 (35.71)	18 (32.14)	
**HCV**				0.2440
Negative	55 (98.21)	32 (57.14)	23 (41.07)	
Positive	1 (1.79)	0	1 (1.79)	
**AFP (ng/mL)**				0.5782
< 400	41 (73.21)	24 (42.86)	17 (30.36)	
≥ 400	14 (25.00)	7 (12.50)	7 (12.50)	
Unknown	1 (1.79)	1 (1.79)	0	
**Cirrhosis**				0.0069
Absent	15 (26.79)	13 (23.21)	2 (3.57)	
Present	41 (73.21)	19 (33.93)	22 (39.29)	
**Clinical stage (BCLC)**				0.4594
A	24 (42.86)	16 (28.57)	8 (14.29)	
B	4 (7.14)	2 (3.57)	2 (3.57)	
C	28 (50.00)	14 (25.00)	14 (25.00)	
**Pathological grading**				0.1877
Well differentiated	11 (19.64)	4 (7.14)	7 (12.50)	
Moderately differentiated	36 (64.29)	24 (42.86)	12 (21.43)	
Poorly & un-differentiated	6 (10.71)	4 (7.14)	2 (3.57)	
Unknown	3 (5.36)	0	3 (5.36)	

Supplementary Table 4. Clinicopathological characteristics of metformin treated HCC patients, according to DOCK1 expression. Related to Fig. 5C

Characteristics	Total No. (%)	DOCK1 expression		
		DOCK1^{Low} No. (%)	DOCK1^{High} No. (%)	*P* value
**Gender**				0.5104
Male	39 (75.00)	16 (30.77)	23 (44.23)	
Female	13 (25.00)	4 (7.69)	9 (17.31)	
**Age at diagnosis, years**				0.0395
< 60	10 (19.23)	1 (1.92)	9 (17.31)	
≥ 60	42 (80.77)	19 (36.54)	23 (44.23)	
**HBV**				0.5802
Negative	18 (34.62)	6 (11.54)	12 (23.08)	
Positive	34 (65.38)	14 (26.92)	20 (38.46)	
**HCV**				0.2015
Negative	51 (98.08)	19 (36.54)	32 (61.54)	
Positive	1 (1.92)	1 (1.92)	0	
**AFP (ng/mL)**				0.5528
< 400	38 (73.08)	14 (26.92)	24 (46.15)	
≥ 400	13 (25.00)	6 (11.54)	7 (13.46)	
Unknown	1 (1.92)	0	1 (1.92)	
**Cirrhosis**				0.2492
Absent	18 (34.62)	5 (9.62)	13 (25.00)	
Present	34 (65.38)	15 (28.85)	19 (36.54)	
**Clinical stage (BCLC)**				0.7807
A	28 (53.85)	12 (23.08)	16 (30.77)	
B	3 (5.77)	1 (1.92)	2 (3.85)	
C	21 (40.38)	7 (13.46)	14 (26.92)	
**Pathological grading**				0.8868
Well differentiated	7 (13.46)	3 (5.77)	4 (7.69)	
Moderately differentiated	36 (69.23)	12 (23.08)	24 (46.15)	
Poorly & un-differentiated	6 (11.54)	2 (3.85)	4 (7.69)	
Unknown	3 (5.77)	3 (5.77)	0	

Supplementary Table 5. Clinicopathological characteristics of HCC patients used for organoid establishment

Patient	Gender	Age (Years)	Type	Liver Disease	Cirrhosis	BCLC	Edmondson	Growth Pattern	AFP (ng/mL)
1	Male	45	HCC	HBV	Yes	C	III	solid	399
2	Female	63	HCC	HBV	Yes	C	III	solid	157119
3	Male	65	HCC	NO	Yes	C	IV	solid	1210
4	Male	70	HCC	HBV	No	-	-	solid	35.51

Supplementary Table 6. Primer sequences used for qPCR

Genes	Species	Forward Primer	Reverse Primer
DOCK1	Homo sapiens	GTTTGCTGCAACCCCTTCTCT	GACCAGCGAACCAGGTAGT
TMED7	Homo sapiens	TCTGGTGGTTAGCATAGGGCA	CAACACGAGTTGTGGTGGTTC
TICAM2	Homo sapiens	AATTCCTGCCCTCTTTCTCTCT	TGCTCAGCAACATTACACAAGG
CMIP	Homo sapiens	CAGCTCACGATTCCTGGGG	CAGCGGCTTGGGTTACTCA
ARPP19	Homo sapiens	AAGGAAACGGTTGCAGAAAGG	GGAGTGGGAATGTGGTCACC
TMEM143	Homo sapiens	GTCCAGGGTCCGAGTATGG	TTCCTGTATTAGGAGGCGGAG

Supplementary Table 7. Antibodies information

Antibody	Company	Catalog Number	Application
RAC1	Abcam	ab33186	Western blot
DOCK1	Proteintech	23421-1-AP	Western blot
DOCK1(H-4)	Santa Cruz	SC-13163	IHC
Ki67(UMAB107)	ZSGB-BIO	ZM-0166	IHC
Ki67(D3B5)	CST	12202T	IHC
Flag(M2)	Sigma	F1804	Western blot
beta-Actin	Proteintech	66009-1-Ig	Western blot
Calnexin	Proteintech	10427-2-AP	Western blot
GST	Proteintech	10000-0-AP	Western blot
YAP1	Proteintech	13584-1-AP	Western blot
phosphotyrosine (4G10)	Millipore	05-1050	Western blot

Supplementary Table 8. Nucleotide sequences of shRNA

Gene	Species	Sequence
DOCK1 sh1	Homo sapiens	CCGGCCGAGCAGTATGAGAACGAAACTCGAGT TTCGTTCTCATACTGCTCGGTTTTT
DOCK1 sh2	Homo sapiens	CCGGCCGAGGTTACACGTTACGAAACTCGAGT TTCGTAACGTGTAACCTCGGTTTTT
DOCK1 sh3'UTR	Homo sapiens	CCGGCATTCTGTACAGAAATTTGTACTCGAGTA CAAATTTCTGTACAGAATGTTTTTG
DOCK1 sh1	Mus musculus	CCGGGCACACGAAGTTGCTTCTAAACTCGAGT TTAGAAGCAACTTCGTGTGCTTTTTG
DOCK1 sh2	Mus musculus	CCGGAGCGATTGGAGCACGTGATTTCTCGAGA AATCACGTGCTCCAATCGCTTTTTTG
